# Significance of image brightness levels for PRNU camera identification

**DOI:** 10.1111/1556-4029.15673

**Published:** 2024-11-19

**Authors:** Abby Martin, Jennifer Newman

**Affiliations:** ^1^ Department of Mathematics Iowa State University Ames Iowa USA

**Keywords:** camera identification, digital forensics, image brightness, image exposure, PRNU, questioned images, source identification

## Abstract

A forensic investigator performing source identification on a questioned image from a crime aims to identify the unknown camera that acquired the image. On the camera sensor, minute spatial variations in intensities between pixels, called photo response non‐uniformity (PRNU), provide a unique and persistent artifact appearing in every image acquired by the digital camera. This camera fingerprint is used to produce a score between the questioned image and an unknown camera using a court‐approved camera identification algorithm. The score is compared to a fixed threshold to determine a match or no match. Error rates for the court‐approved camera‐identification PRNU algorithm were established on a very large set of image data, making no distinction between images with different brightness levels. Camera exposure settings and in‐camera processing strive to produce a visually pleasing image, but images that are too dark or too bright are not uncommon. While prior work has shown that exposure settings can impact the accuracy of the court‐approved algorithm, these settings are often unreliable in the image metadata. In this work, we apply the court‐approved PRNU algorithm to a large data set where images are assigned a brightness level as a proxy for exposure settings using a novel classification method and then analyze error rates. We find statistically significant differences between error rates for nominal images and for images labeled dark or bright. Our result suggests that in court, the error rate of the PRNU algorithm for a questioned image may be more accurately characterized when considering the image brightness.

**Highlights**

- Current error rates for camera identification do not account for image brightness levels.
- The brightness level in an image is labeled by pixel intensity and is consistent with human interpretation.
- Statistically different error rates occur for images with different brightness levels.

## 1 INTRODUCTION

Consider an illicit image at the center of a criminal investigation. One of the most important questions to an investigator is where this image originated. Photo response non‐uniformity (PRNU) source‐camera identification utilizes a pattern of persistent deviations in pixel response across a camera's sensor as a fingerprint to pair a questioned image with the specific camera that took it. In order for a technique to be admitted as evidence in court, an error rate must be associated with the technique's results [1]. False‐positive rates (FPRs) are particularly important in the field of digital forensics because these errors are associated with wrongful convictions when an identification algorithm incorrectly identifies a suspect as the source of the evidence sample. False‐negative rates (FNRs) also have practical implications for forensic practitioners and increase the risk of exonerating the perpetrator of the crime by concluding the suspect is not the source of the evidence sample. Generating accurate error rates facilitates an improved justice system by providing jurors the tools to better weigh the strength of the evidence.

Our previous work [2] provided an initial investigation of the impact of off‐nominally exposed questioned images (too dark or too bright) on PRNU source‐camera identification. We used a carefully collected dataset of 8400 images representing auto‐, under‐, and over‐exposure settings [3]. The errors for the off‐nominal exposure questioned images resulted in FNRs as high as 17.1% and FPRs as high as 0.54% for camera fingerprints estimated from auto‐exposed images. Both error rates are higher than the baseline results using auto‐exposed images in our work (0% FNR and 0.08% FPR in [2]), and higher than the large‐scale test that established the error rates that have been used in court (2.38% FNR and 0.0024% FPR) [4, 5, 6].

However, questioned images will not have an auto‐exposure reference image to determine relative exposure type, as was done in our previous paper. Additionally, EXIF information is easily manipulated, this information may be missing from or unreliable in an evidence image. Therefore, we need a new way to use the image feature of overall brightness to label questioned images as nominal, dark, or bright, which may affect the PRNU source‐camera identification error rates. Such a labeling could provide feature‐sensitive error rate estimates that more accurately reflect the error rates for the case at hand.

To solve the labeling problem, we introduce a novel algorithm that labels the brightness level of a questioned image as nominal, dark, or bright based on only the histogram of pixel intensities in an image, and apply the labels to all images in both datasets. For our labeled images, we show that false‐positive and false‐negative error rates of the PRNU source‐camera identification algorithm are different for off‐nominal (dark or bright) images than for nominal images, and present hypothesis tests that support our findings. We use hypothesis tests to determine the statistical significance of the differences between nominal and dark or bright questioned image error rates, demonstrating that the observed differences are not due to chance [7]. Our hypothesis tests do not address the common‐source problem which has been criticized in forensic applications [8, 9, 10], but instead we use hypothesis tests to validate the forensic algorithm performance for different image brightness levels. We isolate the feature of image brightness by comparing only error rates of images from the same population (e.g., StegoAppDB and Flickr image error rates are estimated separately). Using roughly 60,000 images from two different data sets, we calculate over 8.5 million similarity scores and apply the court‐approved PRNU algorithm to brightness‐labeled images, making this one of the largest studies of PRNU source‐camera identification to date. Additionally, our sample sizes are sufficiently large to observe if an effect of image brightness on the error rates is likely.

The paper is organized as follows: We first detail the problem we aim to address and our overall approach (Section III—Problem and Approach); followed by terminology we use for image brightness or exposure throughout the paper (Section IV—Notation and Terminology). Next, we provide an overview of the development of PRNU source‐camera identification and methodologies to label brightness levels; then we describe the two datasets used in our experiments (Section VI—Data). We then explain and justify our histogram‐based labeling (Section VII—Brightness Labeling Models), describe our experiments (Section VIII—Methods), and explain the hypothesis tests we use to interpret the strength of our results (Section IX—Hypothesis Tests). Finally, we present the results (Section X—Results), the limitations of our experiments and results (Section XI—Limitations), and a discussion of our results (Section XII—Discussion) and conclusions (Section XIII—Conclusion).

## 2 PROBLEM AND APPROACH

Accurate error rates are an essential part of a well‐functioning justice system. We know from prior work [2] that off‐nominally exposed images can have an impact on error rate estimates when using the PRNU source‐camera‐identification algorithm. We propose a histogram‐based brightness labeling to further improve error rate estimates for questioned images with unknown exposure settings by first labeling each image, then applying the PRNU source‐camera‐identification algorithm and finally analyzing the results for the labeled images. We use two datasets, an expanded set of carefully collected images [3] and a set of unknown images from Flickr [11]. The PRNU source‐camera‐identification algorithm [4] is applied to both datasets to determine how bright and dark images impact error rate estimates. To the best of our knowledge, no prior work has identified or proposed a solution to label images with their brightness level to explore the performance of the PRNU source‐camera identification algorithm. Our intention is not to propose new error rates to be used in court, but to show that the error rates for bright and dark images are different from error rates for nominal images. We use hypothesis tests to verify the statistical significance of the difference between error rates for nominal and off‐nominal questioned images.

## 3 NOTATION AND TERMINOLOGY

**FIGURE 1:**
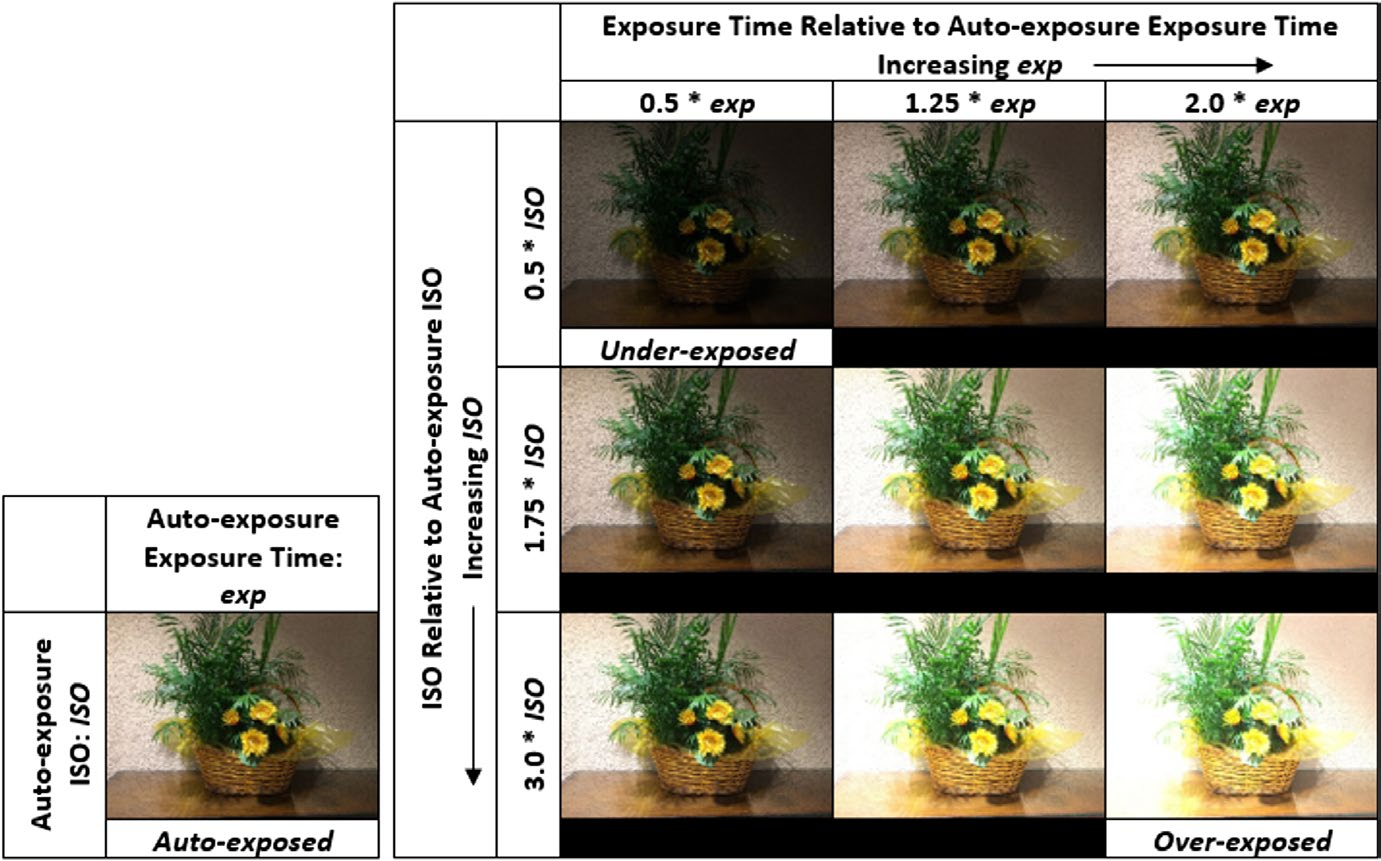
StegoAppDB Images: An example of the nine manual exposure settings (images in the 3 × 3 grid) collected for one StegoAppDB scene relative to the auto‐exposure settings (image on the left‐hand side of the figure). All scenes follow this collection procedure to capture ten images (one auto‐exposed and nine with the manual exposure settings relative to auto‐exposure, as shown).

This section presents the fundamental concepts we use in our work; image notation can be found in the accompanying supplemental material for this publication in the section “Mathematical Notation”. We define specific brightness‐related terms we use in this work: “auto‐exposed,” “under‐exposed,” “over‐exposed,” and “nominal,” “dark,” and “bright.” The terms *auto‐exposed*, *under‐exposed*, and *over‐exposed* are used to reference only particular images from StegoAppDB [3] which are defined by the intentional exposure settings used for data collection. We do not use the terms “auto‐exposed,” “under‐exposed,” or “over‐exposed” for images in the Flickr dataset. An *auto‐exposed* image is one in which the auto‐exposure settings of the camera are used to capture the image, producing an ISO value of ISO and an exposure time of t (see Figure 1 for all calculations for exposure settings relative to the auto‐exposure). The under‐exposed and over‐exposed images are captured relative to the auto‐exposed image, with ISO and exposure times offset from the auto‐exposure settings. The *under‐exposed* image is captured using the auto‐exposed image settings to select the under‐exposed ISO and exposure time, with an ISO of 0.5×ISO and an exposure time of 0.5×t. Similarly, the *over‐exposed* image is captured using the auto‐exposed image settings to select the over‐exposed ISO and exposure time, with an ISO of 3.0×ISO and an exposure time of 2.0×t.

The terms *nominal*, *dark*, and *bright* are class labels output from the classification method for estimating the brightness level for an image that we introduce in Section VII‐B—CCMF Labeling. The parameters for these labels are determined by analyzing the CCMFs for the set of auto‐, under‐, and over‐exposed images in StegoAppDB. Once the parameters are determined from this subset of StegoAppDB data, we produce labels of nominal, dark, and bright for *all* images from StegoAppDB (including the auto, under‐, and over‐exposed images) and the Flickr dataset. The goal of producing a brightness label for an image is to label the image without relying on exposure settings relative to auto‐exposure (which is likely unknown in practice).

## 4 BACKGROUND AND RELATED WORK

There are two foundational areas of research that have influenced this work: PRNU source‐camera identification (Section V‐A—PRNU Source‐Camera Identification) and labeling of off‐nominally exposed images. This section provides an overview of the development of PRNU source‐camera identification. We then discuss papers which label or segment images for applications such as nighttime driving, auto‐exposure, or image segmentation algorithms.

### 4.1 PRNU source‐camera identification

Photo response non‐uniformity (PRNU) refers to a pattern of pixel intensity deviations from the mean response that are due to manufacturing imperfections in the camera's sensor. The PRNU is part of the sensor's fixed pattern noise and is both unique and persistent, making the PRNU an ideal candidate for the camera sensor's fingerprint. This fingerprint is consistently present in every image captured by the camera and allows an image to be matched with the camera that took it. In digital image forensics, we refer to the image of unknown origin as a *questioned image* and the camera under test as the *specific camera*. PRNU source‐camera identification aims to answer whether the specific camera captured the questioned image.

While PRNU‐based source‐camera identification was first introduced in 2005 [12], the algorithm was later standardized in a 2009 large‐scale study on over one million images downloaded from Flickr [4], which has been used in testimony for a federal court case [5, 6]. This PRNU source‐camera‐identification algorithm estimates the PRNU noise of a single questioned image by first applying a Daubchies wavelet‐denoising filter, F, to an image, I, resulting in a noise estimate [13]:

(1) $$W=I-F\left(I\right).$$

Additional image processing, G, is applied to the noise estimate, W, to remove non‐unique artifacts due to model‐specific noise and JPEG compression. Therefore, the questioned image fingerprint, Q, is estimated as [2],

(2) $$\begin{array}{c} Q=G\left(W\right). \end{array}$$

In addition to estimating the questioned image fingerprint, the PRNU source‐camera‐identification algorithm estimates the PRNU fingerprint of the sensor from the specific camera. We estimate this camera fingerprint using 30 images from the specific camera, $I^{\left(i\right)}$, i=1,2,…,30. First, we produce a noise estimate (as in Equation 1) for each image, $W^{\left(i\right)}=I^{\left(i\right)}-F^{\left(i\right)}$. We use this set of noise estimates to calculate a maximum likelihood estimate of the camera fingerprint, $\hat{K}$, as in [14]:

(3) $$\begin{array}{c} \hat{K}=G\left(\frac{\sum_{i=1}^{N}W^{\left(i\right)}I^{\left(i\right)}}{\sum_{i=1}^{N}{\left(I^{\left(i\right)}\right)}^{2}}\right), \end{array}$$

where $G\left(∙\right)$ is the image processing applied to remove non‐unique artifacts; algebraic operations are calculated pointwise at each pixel location.

Finally, the signed peak‐to‐correlation energy (PCE) ratio is calculated between the image fingerprint and the camera fingerprint, where the signed PCE is defined in Equation 5 of [15]. The PCE is a similarity measurement between the image fingerprint, Q, and the pointwise product of the image and camera fingerprint, $I\times \hat{K}$. The PCE was originally proposed to address the issue of cropped and scaled images [16] and incorporates vertical and horizontal shifts between the camera and image fingerprints to determine the sensor alignment which maximizes the correlation score. We specify the horizontal and vertical shifts we incorporate for different‐sized images in Section VIII—Methods. If the PCE score is above 60, we conclude the questioned image is from the specific camera. If the PCE score is at most 60, we conclude the questioned image is not from the specific camera. This threshold of 60 was determined on the set of over one million images from Flickr [4]. The original large‐scale study purports an FNR of 2.38% and an FPR of 0.0024% [4], error rates which the FBI has cited to justify the use of their camera‐identification software [5]. We implement the PRNU source‐camera identification algorithm as described in this section. See [2] for more details.

Previous work has investigated PRNU source‐camera identification error rates for a variety of image features, including JPEG compression [15], vignetting [17], color saturation [18], proprietary image processing or editing software [19, 20, 21, 22, 23], radial distortion corrections [24], and image corrections and enhancements [25]. ISO has been found to impact correlation‐based PRNU source‐camera identification and forgery detection [26, 27, 28], and our previous paper investigates the impact of ISO and exposure time on the PCE‐based algorithm [2]. Additionally, one work questioning the uniqueness of the PRNU camera fingerprint using a set of over 30,000 images from Flickr found false‐positive error rates as high as 99.2% [11]. None of these prior works use a dark or bright labeling as part of the method to determine PRNU error rates.

Once we compute error rates, we present hypothesis tests to determine whether the error rates we calculate from our labeled images differ between nominal and off‐nominal (dark or bright) images. The source‐camera identification problem was formulated as a hypothesis test as early as [13] and in the foundational 2009 large‐scale study [4]. Both papers pose a null hypothesis that the questioned image is from some unknown camera and an alternative hypothesis that the questioned image is from the specific camera, which avoids the pitfall of assuming a match which is common in forensic hypothesis tests. Neither paper presents a p‐value that allows the interpretation of the probability of the null hypothesis or the determination of the statistical significance of the results. Instead, the results in [4] rely on the PCE score in relation to a threshold with a bounded false‐positive rate to make a match or non‐match decision.

Hypothesis tests have had a rocky road in forensic science. While the question of guilt or innocence may seem ideal for the hypothesis‐test framework, the p‐value interpretations for these hypothesis tests are often misunderstood and overstated [8, 9]. The field of forensic glass comparison has asked a slightly different question. Forensic glass comparison has used hypothesis tests, not to determine guilt or innocence of a defendant, but to determine whether evidence glass samples and known samples share an origin [10]. This is based on the relative concentration of elements in each sample and whether the differences between the two samples could be due to chance. However, this common‐source question is still flawed, as the burden of proof shifts to the defendant by assuming a null hypothesis that the evidence and known sample are from the same source. Our hypothesis tests do not aim to determine the source question (i.e., whether an image was taken by a specific camera), but instead focus on whether the differences in the error rates of PRNU source‐camera identification between nominal and off‐nominal images are due to chance [7]. This type of hypothesis test can be viewed as exploring a bias that may be inherent in an algorithm by examining the algorithm's output on data having different feature values. An example of the identification of the bias in an algorithm is the examination of race in recidivism prediction algorithms. Hypothesis tests provided overwhelming evidence that recidivism prediction algorithms treated Black and White inmates differently, indicating bias in these algorithms that could influence parole recommendations [8]. In our case, we ask the question, do PRNU error rates differ for images that are labeled with different feature values of brightness? We use a hypothesis test to validate the performance of the error rate estimates for the PRNU source‐camera identification algorithm on sets of images with different feature values for brightness: nominal, dark, or bright.

### 4.2 Image brightness labeling

There has been limited research whose goal is to classify the entirety of an image as nominal, dark, or bright. Instead, many works focus on identifying regions in an image where the local brightness level makes objects or details of the image poorly recognizable due to lack of contrast. Once an image is enhanced, continued processing toward the main research goal proceeds. There are many applications where the enhancement of images is important, although the majority focus on under‐exposed images and their downstream processing. Most works create a procedure that takes an input image that is poorly exposed or has many regions poorly exposed and outputs an enhanced version that has a visually pleasing brightness level. No works, to the best of our knowledge, provide a specific algorithm to produce an overall brightness classification of an image. There are too many works to cite here, but a few very common applications include nighttime driving [29, 30]; brightness correction of over‐ and under‐exposed images [31]; object recognition [32]; video enhancement [33]; and image segmentation [34].

In addition to creating our own novel classification algorithm to label nominal, dark, or bright images, we validate the class assignments using several independent verifications. Other datasets containing dark or bright images also validate their data. In [32], the authors collect 7363 Internet images using humans to decide the level of darkness for their “low‐light data.” The authors in [29] collect videos from a car during specific times of the day, twilight through nighttime, and extract frames from the videos, thus using time‐of‐day to produce the lightness labeling of the image. The authors in [31] use the raw‐RGB images in the MIT‐Adobe FiveK dataset and adjust those images to realistically emulate different camera exposure values, producing over or under‐exposed images. The validation of their data is done using Adobe CameraRaw's camera rendering procedure (formulaic). One last type of verification is to use the histogram of an image to determine threshold values that give low error rates when used for user authentication [35], or to maximize interclass variance between dark and bright regions for object recognition [34].

Part of the work in [35] is the closest to our labeling method, providing some results on dark and bright images for their authentication procedure. The PRNU camera sensor fingerprint is used as a hardware fingerprint to deter hackers from using contrived fingerprints as a user authentication attack on a smartphone. Their goal is to determine key factors that produce reliable PCE matches between an image assumed to be from the smartphone and a stored reference fingerprint for the smartphone. They report that very dark images and very bright images, among other factors, produced poor PCE values.

Due to the lack of a standard classification for overall image brightness level—nominal, dark, or bright—we propose a classification method based on the CCMF of an image's pixel intensities in Section VII‐B—CCMF Labeling and provide validation of the labels for our datasets in Section VII‐C—Human Judgment.

## 5 DATA

Our experiments are performed on two datasets: StegoAppDB [3], and a set of over 30,000 images from Flickr from Iuliani et al. [11]. The two datasets are kept separate for the purposes of our experiments and results, but provide insight into the impact of the data collection protocol. We provide an overview of the images from each dataset, with StegoAppDB in Section VI‐A—StegoAppDB Details and the Flickr data in Section VI‐B—Flickr Data. In addition, we provide justification for utilizing our auto‐, under‐, and over‐exposed images as ground truth for nominal, dark, and bright images, respectively, by three independent methods: (1) exposure settings; (2) exposure values; and (3) human judgment.

### 5.1 StegoAppDB details

StegoAppDB [3] is a carefully collected dataset of images with ten different exposure settings relative to the auto‐exposure for each image from twenty‐eight smartphone cameras, shown in Table 1. The ten exposure settings consist of an auto‐exposure image and nine manual exposure settings relative to the auto‐exposure settings for each specific scene collected, as shown in Figure 1. For the auto‐exposure ISO value of each scene, ISO, we calculate three manual ISO settings: 3.0×ISO, 1.75×ISO, and 0.5×ISO. For the auto‐exposure time of each scene, exp, we calculate three manual exposure time settings: 2.0×exp, 1.25×exp, and 0.5×exp. We collect the nine manual exposure images relative to each auto‐exposed image by iteratively combining the three manual ISO settings and the three manual exposure time settings using either an Android app (developed with Java) or an iOS app (developed with Swift) called Cameraw.

**TABLE 1:** The camera models, operating systems, and number of devices for each model in StegoAppDB. The auto‐exposed and nine manual exposure settings are captured for each scene from the 28 smartphone cameras.

Camera model	Operating system	Number of devices
Pixel1	Android	4
Pixel2	Android	4
iPhone6s	iOS	4
iPhone7	iOS	4
iPhone8	iOS	2
OnePlus5	Android	2
SamsungS8	Android	2
iPhone6sPlus	iOS	2
iPhone7Plus	iOS	2
iPhoneX	iOS	2

There are two data collection protocols for StegoAppDB: same scene and unique scene. The eighteen cameras that follow the same‐scene protocol (first 5 models in Table 1) capture images with the same set of scene content, as the images were taken sequentially by replacing the camera on a stationary tripod. Approximately 100 scenes were captured for these 18 cameras. The remaining ten cameras follow the unique‐scene protocol and collected their images independently of one another and, therefore, do not repeat scene content. Approximately 100 scenes were captured per camera for these models. We guarantee that no questioned image shares scene content with an image used to estimate its specific camera's fingerprint.

Of the 28,360 StegoAppDB images we use for experiments in this paper, we call the set of auto‐exposed images for all cameras A where A has 2386 images. Similarly, we call the set of under‐exposed images U, where U has 2836 images, and the set of over‐exposed images O, where O has 2836 images. The terms auto‐, under‐, and over‐exposed images A∪U∪O are used as ground‐truth image classes for nominal, dark, and bright, respectively, in the brightness classification method. The remaining seven manual‐exposure images from StegoAppDB are not labeled as auto‐, under‐, or over‐exposed. Instead, they are assigned labels according to the brightness classification method.

The rest of this section is used to present justification and validation of the use of the sets A∪U∪O for ground‐truth brightness labels. We feel it is important to validate the definition of *exposure* we use in our work to label our data. This is because interpreting the error rates produced by the PRNU camera identification algorithm on the separate classes depends on the labels of the data. To this end, we provide three different ways to validate the exposure types of the ground truth data in StegoAppDB: exposure collection settings; an interrater agreement study with human judges; and exposure values. While we do not use the EXIF or metadata to label the exposure of unknown images, we do use the exposure settings from the set A∪U∪O in StegoAppDB as ground truth for the three exposure types when we determine the parameters for our classification method.

In addition to the collection settings detailed above, a second validation for the brightness labels for A∪U∪O is an interrater agreement study. This interrater agreement study between three human judges and the computer program, performed in our prior work [2], found a near‐perfect level of agreement between the four sets of responses, supporting our claim that the auto‐, under‐, and over‐exposed images correspond to our visual expectations of such exposure types.

Our third validation for the labels of the exposure types for A∪U∪O applies the definition of photographic exposure value. Exposure value (EV) is a single number combining the camera's f‐number (related to the aperture) and exposure time t. The EV number represents the many combinations of f‐number and exposure time quantifying the same amount of light on the sensor, with common values ranging from negative values (−7 for a night scene) to 16 (a bright snowy picture). For our data, we assume scenes are subjected to typical indoor light distributions. The camera's auto‐exposure program mode chooses optimal exposure settings to produce a pleasing picture, not too light or too dark. We view the EV number that corresponds to the auto‐exposure setting of a camera as an intrinsic property of a pleasing photographic scene acquired at a specific time, location, and lighting condition. The details of the EV for the auto‐, under‐, and over‐exposed images can be found in the accompanying supplementary material. Note that an under‐exposed image has an EV strictly less than auto‐exposed images and an over‐exposed image has an EV strictly greater than auto‐exposed images for the StegoAppDB dataset.

The exposure values for the under‐ and over‐exposed StegoAppDB images relative to their corresponding auto‐exposed image provide a third validation for our use of these images as ground truth dark and bright images, respectively. This set of auto‐, under‐, and over‐exposed images are used as ground truth to determine parameters for our CCMF classification method proposed in Section VII‐B—CCMF Labeling. Our intentionally different exposure values delineate our research from prior work investigating the impact of ISO [28], where the authors changed the exposure time to maintain a constant exposure value for different ISO settings.

### 5.2 Flickr data

**FIGURE 2:**
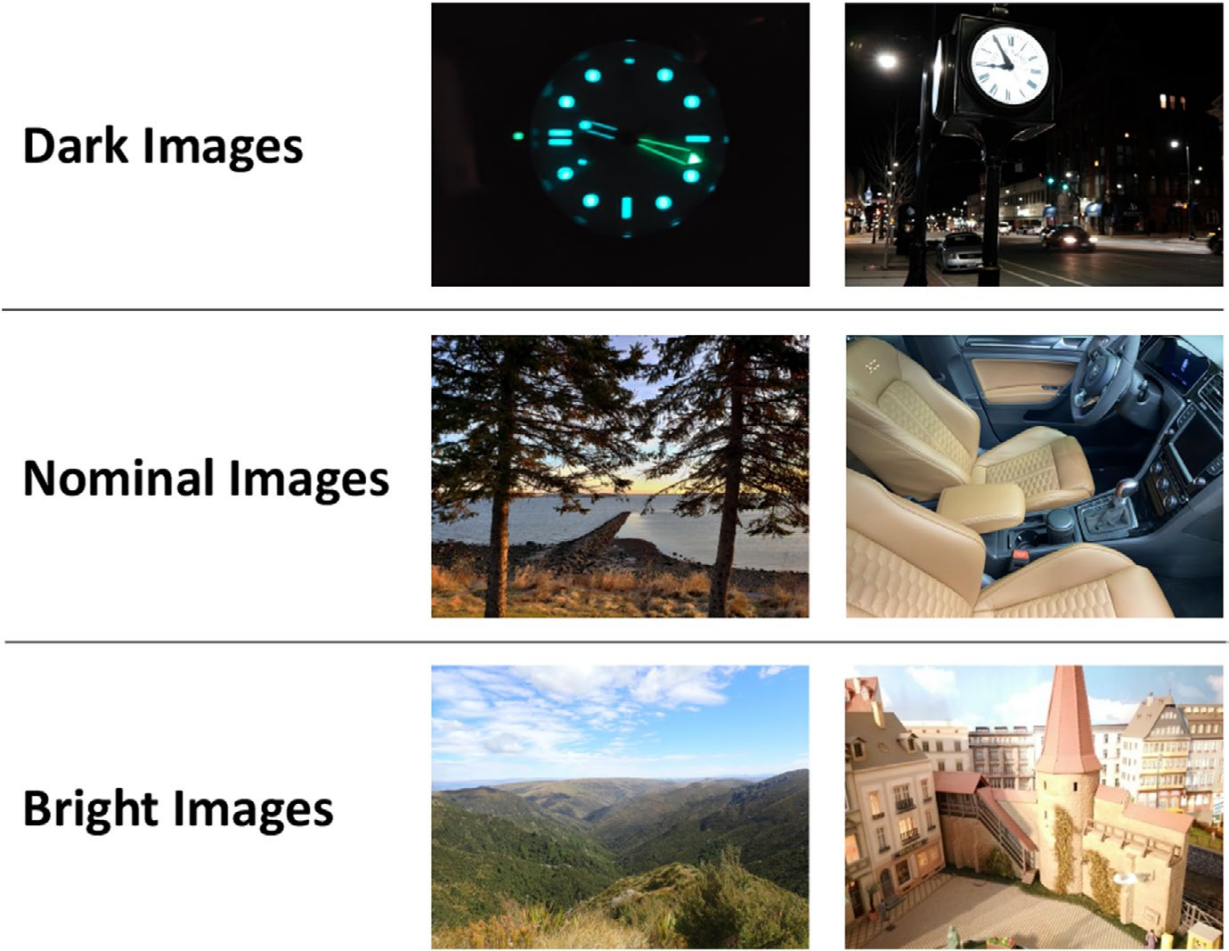
Flickr Images: Two examples of nominal, dark, and bright images from the Iuliani et al. Flickr dataset labeled using our classification method.

While StegoAppDB is a dataset comprising carefully collected images, the second dataset we use is likely more representative of true questioned images. The 31,994 images from Flickr provided by Iuliani et al. [11] were uploaded to Flickr by the website's users. The Iuliani Flickr dataset [11] more closely aligns with the collection of a questioned image, except for some biases inherent in uploading your “personal best” images to a public website. This Flickr dataset consists of 495 cameras representing 79 camera models. Of these, 120 cameras are traditional point‐and‐shoot cameras representing 10 brands (e.g., Canon or Nikon) and 31 camera models. An additional 375 smartphone cameras are from 9 brands (e.g., Apple or Samsung) and 48 camera models. Only 340 of the 495 cameras have at least 30 nominal images to estimate the camera fingerprint. Therefore, we estimate only 340 camera fingerprints for the Flickr data, using a total of 10,200 Flickr images. Additionally, some cameras only have the 30 nominal images and, therefore, do not have any questioned images from the camera, and thus, these other cameras are only used to calculate false‐positive rates in our experiments. There is a total of 21,794 questioned images from 482 cameras. Due to the lack of a data collection protocol for taking the pictures, no images are omitted due to shared scene content with an image used to estimate the camera fingerprint, as is the case for the StegoAppDB images. We show two examples each of nominal, dark, and bright images from Flickr that are labeled using our classification method in Figure 2.

## 6 BRIGTHNESS LABELING MODELS

To produce a reliable classification method for labeling an image as nominal, dark, or bright, we investigate several machine learning models and a CCMF‐based thresholding method. The CCMF classification method to label brightness levels finds optimal parameters using the CCMFs from auto‐, under‐, and over‐exposed images in StegoAppDB to produce the image labels nominal, dark, and bright.

### 6.1 Machine learning methods

Our machine learning models are trained and tested using the 8508 images in A∪U∪O from StegoAppDB. We assume the auto‐exposed images are nominal, the under‐exposed images are dark, and the over‐exposed images are bright and attempt to classify the images accordingly. Four common machine learning models (decision trees, random forests, support vector machines, and convolutional neural networks) provide a comparison with our proposed CCMF classification method. For each of the following models, 50% of the data is used for training and 50% of the data is used for testing. The decision trees, random forests, and support vector machines each have an accuracy of 100% for labeling nominal, dark, and bright images from StegoAppDB. The two CNNs have accuracies of 96.50% and 96.36%. Details of the model implementation for the decision trees, random forests, support vector machines, and CNNs can be found in the accompanying supplement material. Machine learning models are commonly utilized for classification problems and, therefore, serve as an intuitive method for labeling nominal, dark, and bright images.

### 6.2 CCMF labeling

**FIGURE 3:**
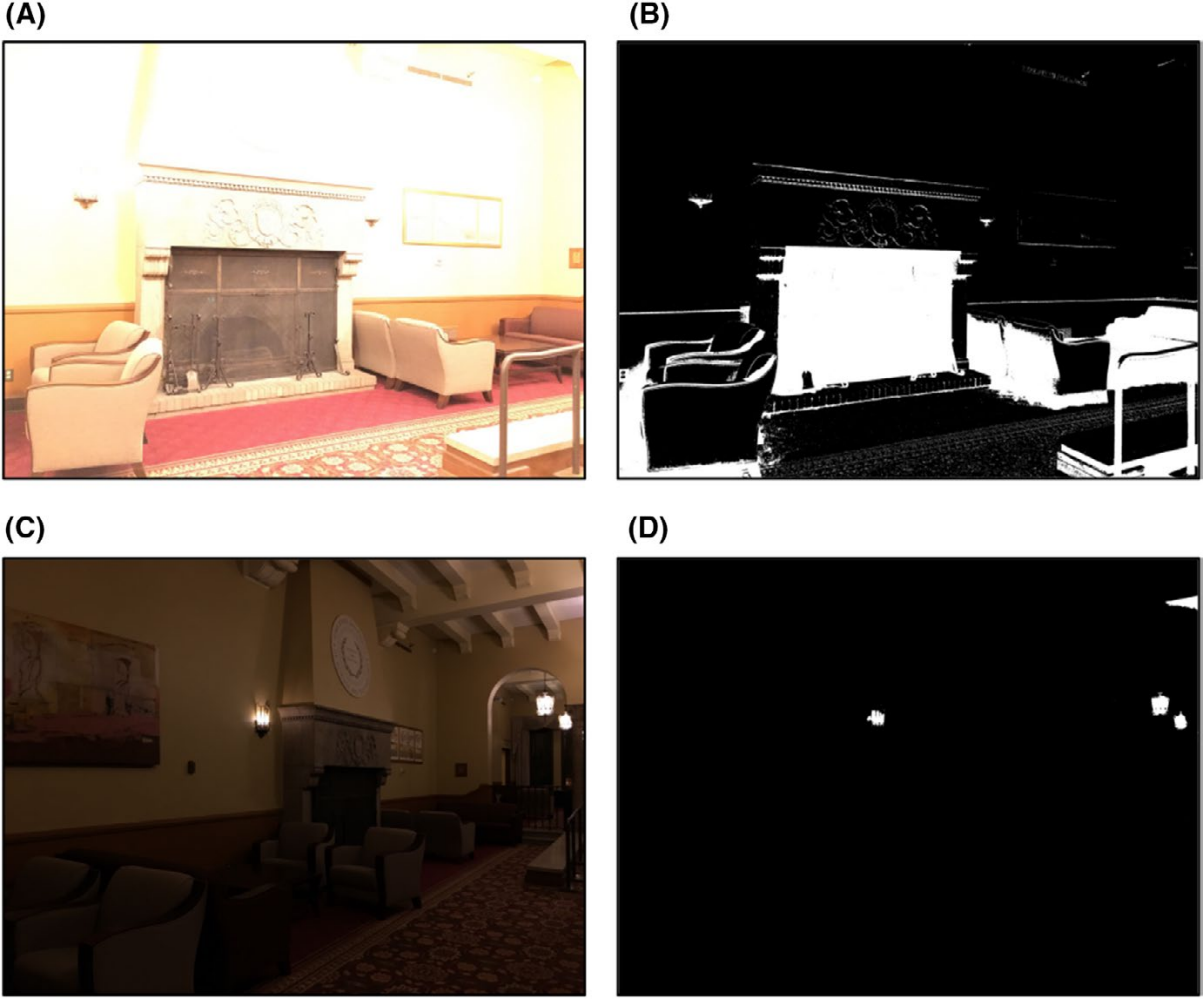
Area in image with high or low pixel intensities. (A) Original bright image. (B) Image (A) with pixels with intensity values ≥ 238 turned black and all other pixels turned white (about 80.16% of the total image). (C) Original dark image. (D) Image (C) with pixels with intensity values less than 149 turned black and all other pixels turned white (about 99.66% of the total image).

While the machine learning methods were quite accurate, we ultimately decided to implement a classifier for nominal, dark, and bright using a threshold applied to the image CCMF. We base our classification method on the simple (and general) observation: a dark image has more pixels with low‐intensity values than a nominal image. Similarly, a bright image has more pixels with high‐intensity values than a nominal image. See Figure 3 for an example. In Figure 3 (B), the black regions are the pixel locations of the bright image in (A) whose pixel intensities are at least 238 (about 80.16%). In Figure 3 (D), the black area is the pixel locations of the dark image in (C) whose pixel intensities are less than or equal to 149 (about 99.66%). We use the set of StegoAppDB auto‐, under‐, and over‐exposed images as ground‐truth nominal, dark, and bright images, respectively, to find parameters for the classification method and then apply it to the entire StegoAppDB and Flickr datasets. This includes attaching a (potentially new) class label to the original ground‐truth images for auto‐, under‐, and over‐exposed images. Note that the under‐ and over‐exposed images in StegoAppDB are purposefully collected to be extremely dark or extremely bright, meaning that the labeled nominal images cover a wide range of brightness levels. We analyze the differences between the CCMFs of the auto‐ and under‐exposed images in StegoAppDB to determine threshold values above which an image is classified as “nominal” and below which an image is classified as “dark.” We can calculate the dark accuracy, ${\mathrm{Acc}}_{D}\left(k,f\right)$, for each k, pixel intensity threshold, and f, fraction of image pixels, pair used to separate the dark and nominal images.

Our goal is to find a gray value ${\hat{k}}_{D}$ and a fraction ${\hat{f}}_{D}$ that provide threshold values for a pixel intensity and the fraction of intensity values, respectively, that maximize the classification accuracy of A∪U into the two classes of “nominal” and “dark” using the image set A∪U as training data. To this end, we simply run an exhaustive search on the 256×101 discrete grid $\left\{\left(k,f\right):0\leq k\leq 255,f=0,0.01,0.02,\ldots ,1.0\right\}$ and compute the accuracy for each grid point. We find that for the dark accuracy,

(4) $$\begin{array}{c} \left({\hat{k}}_{D},{\hat{f}}_{D}\right)=\underset{\left\{\left(k,f\right)\right\}}{\text{argmax}}\left\{{\mathrm{Acc}}_{D}\left(k,f\right):0\leq k\leq 255,f=i*0.01,i=0,\ldots ,100\right\}=\left(149,0.07\right). \end{array}$$

**FIGURE 4:**
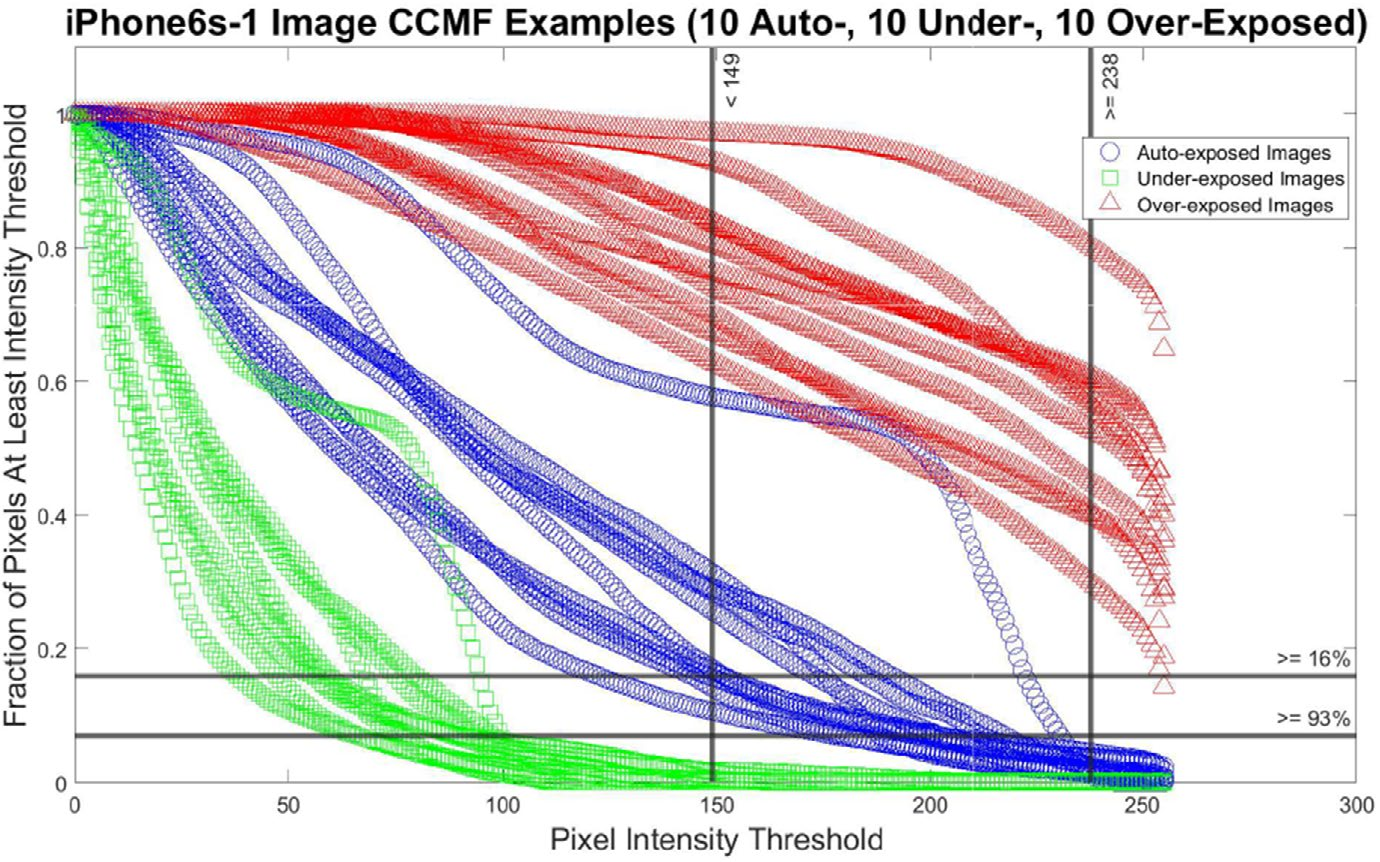
CCMF Examples: The CCMFs for 10 auto‐ (blue lines), 10 under‐ (green lines), and 10 over‐exposed (red lines) iPhone6s‐1 images from StegoAppDB. If at least 93% (lower horizontal black line) of the pixel intensities are at most 149 (left‐hand vertical black line), then the image is labeled “dark.” If at least 16% (upper horizontal black line) of the pixel intensities are at least 238 (right‐hand vertical black line), then the image is labeled “bright.” Otherwise, the image is labeled “nominal.”

This result states that the paired values $\left(k,f\right)$ that provide the division of auto‐ and under‐exposed images into nominal and dark groups with the least error occur when ${\hat{k}}_{D}=149$ and ${\hat{f}}_{D}=0.07$. The maximum dark accuracy value of ${\mathrm{Acc}}_{D}\left(k,f\right)=98.94\%$ results when an image I is labeled as “dark” if $S^{I}\left(149\right)<0.07$, or, equivalently, if at least 93% of its pixel intensities are less than 149. An image is labeled as “nominal” if $S^{I}\left(149\right)\geq 0.07$, if less than 93% of its pixel intensities have intensity values at most 149. Only ten auto‐exposed images are labeled “dark” and fifty under‐exposed images are labeled “nominal.” Examples of CCMFs for nominal and dark images from an iPhone 6 s are shown by the blue and green lines in Figure 4, respectively.

We do a similar process using the CCMFs for auto‐ and over‐exposed StegoAppDB images. Calculations show that the solution to this optimization problem is ${\mathrm{Acc}}_{B}\left(238,0.16\right)=99.96\%$. This is interpreted as follows: An image I is labeled “bright” if $S^{I}\left(238\right)\geq 0.16$. An image is labeled “nominal” if $S^{I}\left(238\right)<0.16$. Only one auto‐exposed image is mislabeled “bright” and one over‐exposed image is mislabeled “nominal.” Examples of CCMFs for nominal and bright images from an iPhone 6 s are shown by the blue and red lines in Figure 4, respectively.

In conclusion, we label an image “dark” if at least 93% of the pixel intensities of the image are less than 149 and label an image “bright” if at least 16% of the pixel intensities of the image are greater than or equal to 238. Otherwise, an image is labeled “nominal.” The overall accuracy of our classification method for labeling the set A∪U∪O as nominal, dark, and bright is 99.27%, with a total of 62 out of 8508 images mislabeled. Of the 28,360 images in StegoAppDB, 13,979 are labeled nominal, 3257 are labeled dark, and 11,124 are labeled bright. Of the 31,994 Flickr images, 29,690 are labeled nominal, 1352 are labeled dark, and 952 are labeled bright. This error rate is comparable with the results from our machine learning classification methods (100% accuracy for the three machine learning models compared with an accuracy of 99.27% for our CCMF labeling method). We remark that while the CCMF classification does not have 100% accuracy as the machine learning models do, there are two advantages: first, the labeling method is easier to explain to a jury in court than a machine learning method. Second, if the characterizations for the three brightness level classes change due to a different training dataset, or a different brightness labeling definition, the optimization procedure is quick and easy to implement.

### 6.3 Human judgment

A validation of our image brightness CCMF classification method measures the agreement between three human judges and our classification method's characterization of nominal, dark, and bright images. If the labeling consistency between these four responses is high enough, we are satisfied that our threshold‐based labeling satisfactorily characterizes nominal, dark, and bright images. A high level of agreement between the human and CCMF labelings demonstrates our new labeling scheme is in‐line with the characterizations that a human observer would expect and provide a logical check on our methodology. We perform four validation tests: (1) nominal versus bright StegoAppDB images, (2) nominal versus dark StegoAppDB images, (3) nominal versus bright Flickr images, and (4) nominal versus dark Flickr images.

For StegoAppDB validation, we used the 13,979 “nominal,” 3257 “dark,” and 11,124 “bright” labeled images. This set of images is too large for a human judge to complete a labeling in a reasonable amount of time without fatigue, so we randomly selected 250 off‐nominal images for a human‐judgment study. Half of those (120 images) were too dark and the other half were too bright. The set of 120 dark images was mixed with 125 randomly drawn, nominally exposed images to form a set of 250 images. This process of mixing in 125 randomly drawn nominal images was repeated for the bright images, resulting in a second set of 250 images.

The two sets of 250 images were shown to human judges using Google Forms to poll the responses. The judge clicked one of three text boxes indicating their judgment as “too light,” “properly exposed,” or “too dark.” When a text box was clicked, the tool logged the choice.

The resulting data were analyzed using Fleiss's kappa [36] with four raters—three human judges and the computer program that chose the images in the first place. Using the “*R*” statistical software package *irr* and the “*R*” function *kappam.fleiss* [37, 38], we calculated the kappa values, z‐statistics, and p‐values. The result of each paired test is shown in Table 2.

**TABLE 2:** The results measuring the level of agreement between nominal, dark, and bright labelings by human judges and our classification method. Fleiss's Kappa is used to measure the level of agreement and for each comparison, we provide the Kappa value, the z‐statistic, and the p‐value. The p‐value for each set of data is 0, indicating a nearly perfect agreement among the four raters.

	Kappa value	z‐statistic	p‐value
Nominal versus dark StegoAppDB	0.708708	27.44814	0
Nominal versus bright StegoAppDB	0.7876576	30.44478	0
Nominal versus dark Flickr	0.6806163	26.30738	0
Nominal versus bright Flickr	0.5621722	21.77284	0

We repeat this validation test using the data from Flickr. The Flickr data comprise 29,690 nominal images, 1352 dark images, and 952 bright images. Once again, we select 250 off‐nominal images (half too bright and half too dark) and mix each half with 125 randomly selected nominal images. We use the same Google Form process to obtain results from our three human judges and analyze the responses using Fleiss's kappa. The results are shown in Table 2.

All four validation tests have a p‐value of 0, indicating a nearly perfect level of agreement between the three human judges and our classification method. Therefore, we conclude that our classification method is a reasonable and justifiable choice for separating nominal, dark, and bright images for the images in the StegoAppDB and Flickr datasets.

## 7 METHODS

Once the data labeling is complete, we apply the PRNU source‐camera identification algorithm and analyze the error rates based on the labels of the questioned images. The experiments proposed in this section aim to determine whether PRNU source‐camera identification error rates differ for nominal, dark, and bright images for two different datasets: StegoAppDB and the Flickr dataset. We perform two experiments using StegoAppDB data: one experiment that uses camera fingerprints calculated with auto‐exposed images (see Section VI‐A—StegoAppDB Details) and a second experiment that uses camera fingerprints calculated with images labeled “nominal” by the CCMF algorithm. We perform one experiment using Flickr data: all camera fingerprints are calculated with images labeled “nominal” from the CCMF algorithm. Thus, our experiments are as follows: (1) StegoAppDB: auto‐exposed camera fingerprint versus nominal, dark, or bright questioned images; (2) StegoAppDB: nominal camera fingerprint versus nominal, dark, or bright questioned images; and (3) Flickr data: nominal camera fingerprint versus nominal, dark, or bright questioned images. This results in a total of 8,553,760 PCE scores calculated.

For the three experiments, we do not admit any shifts for our PCE calculation when the questioned image is the same size as the camera fingerprint. When the questioned image is a different size than the camera fingerprint, we admit all shifts. The details of how shifts are performed in the PCE calculation are provided in [4]. All specific‐camera fingerprints are estimated using 30 auto‐exposed (StegoAppDB) or 30 nominal (StegoAppDB and Flickr) images. By calculating the error rates for different populations separately (e.g., we do not compare StegoAppDB error rates with the Flickr error rates), we are able to isolate the impact of image brightness. Additionally, the consistency of the significance of the error rate differences for both datasets contributes to the strength of the evidence that the error rates for dark/bright images differ from the error rates for nominal images. It is not our goal to calculate a universal error rate estimate for any specific type of image but instead to determine whether significant differences exist between error rates of different image types. We present details of these experiments next.

### 7.1 StegoAppDB: Auto‐exposed camera fingerprint

The camera fingerprint for each of the twenty‐eight StegoAppDB cameras (Table 1) is estimated using 30 randomly‐selected auto‐exposed images.

The PCE score is calculated between each of these twenty‐eight auto‐exposure camera fingerprints and 9849 nominal, 2281 dark, and 7830 bright questioned images from StegoAppDB. This results in a total of 28×19,960=558,880 PCE scores. Note that some images are omitted from the questioned image set due to shared scene content with an image used to create the camera fingerprint. Also, note that the orientation of the images is known due to our data collection protocol and, therefore, we do not calculate the PCE score for any rotations of the StegoAppDB images. Of these 558,880 PCE scores, 19,960 are for questioned images that are from the specific camera and contribute to the TPR and FNR. 538,920 PCE scores are for questioned images that are not from the specific camera and contribute to the TNR and FPR. Results for the StegoAppDB auto‐exposed camera fingerprint experiments are shown in Section X‐A—StegoAppDB: Auto‐Exposed Camera Fingerprint.

### 7.2 StegoAppDB: Nominal camera fingerprint

For the second experiment, the camera fingerprints for each of the twenty‐eight cameras in StegoAppDB are estimated using 30 randomly selected nominal images (different from using auto‐exposed images in Section VIII‐A—StegoAppDB: Auto‐Exposed Camera Fingerprint). The PCE scores are calculated between each nominal camera fingerprint and 10,246 nominal, 2418 dark, and 8226 bright questioned images from StegoAppDB, resulting in a total of 584,920 PCE scores. The count of questioned images is different from in Section VIII‐A—StegoAppDB: Auto‐Exposed Camera Fingerprint because the two camera fingerprint image sets are randomly selected, and we remove questioned images with the same scene content as the images used to estimate the camera fingerprint. We do not calculate the PCE score for any rotations of the StegoAppDB images because we used the same camera orientation when collecting these images. From this set of 584,920 PCE scores, 20,890 are for questioned images from the specific camera and 564,030 are for questioned images not from the specific camera. Results are shown in Section X‐B—StegoAppDB: Nominal Camera Fingerprint.

### 7.3 Flickr data: Nominal camera fingerprint

The final experiment calculates the camera fingerprint using 30 randomly selected nominal images for each of the 340 cameras in the Flickr dataset [11] to estimate the camera fingerprint for each of the 340 cameras. We calculate the PCE score between each of the 340 camera fingerprints and the 19,490 nominal, 1352 dark, and 952 bright questioned images. This results in a total of 21,794×340=7,409,960 PCE scores, with 12,740 of these PCE scores corresponding to questioned images from the specific camera and 7,397,220 corresponding to questioned images not from the specific camera. The orientation of the Flickr images is unknown and, therefore, we do calculate the PCE score for rotations of the Flickr images. We calculate the PCE score for the two rigid rotations of the questioned image (either 90 and 270 degrees or 0 and 180 degrees, depending on whether the questioned image and camera fingerprint have the same orientation) and use the larger of the two PCE scores for comparison. The results are presented in Section X‐C—Flickr Data: Nominal Camera Fingerprint.

## 8 HYPOTHESIS TESTS

In order to quantify the strength of our results, we consider the difference in error rates between one type of error (FNR or FPR) and two populations as a hypothesis test. A hypothesis test provides evidence of whether the difference between two error rates (of the same type) is due to chance, or whether the true error rates of the two populations differ [7]. In our case, if the hypothesis test shows that the true error rate for the population of nominal images is different (higher or lower) than the true error rate for the population of one set of off‐nominal images, then the type of error rate estimate for the PRNU algorithm for these two populations must be estimated and presented as separate cases. Therefore, being able to identify an evidence image as “dark” or “bright” indicates that the strength of evidence is different from nominal images. This type of hypothesis test is being used to validate the PRNU algorithm's veracity on images with different brightness levels, not the veracity of the source determination of the PRNU algorithm. Our use of a hypothesis test in this setting is different from other uses where the hypothesis test is constructed to ask: does this piece of evidence originate from this source? We believe this is the first publication using a hypothesis test to test the effect of data features on the error rates, rather than on the source evidence itself. Other uses of source hypothesis testing for forensics can be found in [8, 9, 10].

First, we describe the hypothesis test for the FNR for PCE scores. An error rate is a measure of success and failure, so here we regard correct matches between questioned images and a specific camera (True Positives) as “successes.” Similarly, we consider incorrect non‐matches (False Negatives) as “failures.” Although our questioned images are drawn without replacement, our population size is large enough that the hypergeometric distribution can be approximated using a binomial distribution, as the population of nominal and dark images (and, separately, nominal and bright images) is well over twenty times the size of our samples [39]. Additionally, the images are randomly partitioned into camera fingerprint and questioned image sets; therefore, the images used as questioned images have been randomly selected. There is also no overlap between the nominal, dark, and bright image sets.

Consider the hypothesis test for nominal and dark images. Let $\theta_{N}$ denote the proportion of “successes” for the set of nominal questioned images, and let $\theta_{D}$ denote the proportion of “successes” for the set of dark questioned images. We are interested in whether dark questioned images change the FNR (i.e., change the TPR) and, therefore, utilize a two‐sided hypothesis test. This hypothesis test can be stated:

(5) $$\begin{array}{c} H_{0}:\theta_{N}-\theta_{D}=0 \end{array}$$

(6) $$\begin{array}{c} H_{a}:\theta_{N}-\theta_{D}\neq 0. \end{array}$$

Let ${\hat{\theta }}_{N}$ and ${\hat{\theta }}_{D}$ be proportion estimates from our sample, where $X_{N}$ and $X_{D}$ are the counts of successes for the nominal and dark images, respectively, and $n_{N}$ and $n_{D}$ are the sizes of the two samples. Then:

(7) $$\begin{array}{c} {\hat{\theta }}_{N}=\frac{X_{N}}{n_{N}} \end{array}$$

(8) $$\begin{array}{c} {\hat{\theta }}_{D}=\frac{X_{D}}{n_{D}}. \end{array}$$

Then, the test statistic used to evaluate this hypothesis test is:

(9) $$\begin{array}{c} z=\frac{{\hat{\theta }}_{N}-{\hat{\theta }}_{D}}{\frac{\left(X_{N}+X_{D}\right)}{n_{N}+n_{D}}\left(1-\frac{\left(X_{N}+X_{D}\right)}{n_{N}+n_{D}}\right)\sqrt{\frac{1}{n_{N}}+\frac{1}{n_{D}}}}. \end{array}$$

We then determine the probability of observing z‐statistic as more extreme than the observed value assuming $\theta_{N}$ equals $\theta_{D}$, also known as the p‐value, using a normal distribution. We interpret the strength of the p‐value as evidence for the alternative hypothesis, instead of setting an α‐level of significance to reject the null hypothesis. This interpretation is preferred by the American Statistical Association [40]. However, our hypothesis tests are not independent and, therefore, introduce a problem due to multiple comparisons. We adjust the rejection criterion for each of the individual hypotheses using the Bonferroni method [41], meaning we use a significance level of α/T where T=4 is the number of tests we are performing. Thus, a p‐value of greater than 0.025 indicates little to no evidence, between 0.025 and 0.0125 indicates borderline/weak evidence, between 0.0125 and 0.00625 indicates moderate evidence, between 0.00625 and 0.00025 indicates substantial/strong, and below 0.00025 indicates overwhelming evidence for the alternate hypothesis. We repeat this hypothesis test for each set of experiments for the set of bright questioned images.

We also perform a similar hypothesis test for the FPR, where successes are defined as correct rejections (True Negatives) and failures are incorrect matches of questioned images not from the specific camera (False Positives). The remainder of the hypothesis test remains the same.

In addition to our hypothesis tests, we perform post hoc power analysis for the non‐significant results. A power analysis is an indication of the likelihood of committing a Type II error—failing to observe an effect when, in reality, the effect does exist. The objective of the power analysis is to determine whether the sample size is large enough to observe an effect for the feature under test (either dark or bright images). We acknowledge that post hoc power analyses are largely dependent on the p‐value of the hypothesis test and may not add notable results [42, 43]. However, we include this analysis for completeness and as a possible explanation if an effect was not observed.

## 9 RESULTS

### 9.1 StegoAppDB: Auto‐exposed camera fingerprint

**FIGURE 5:**
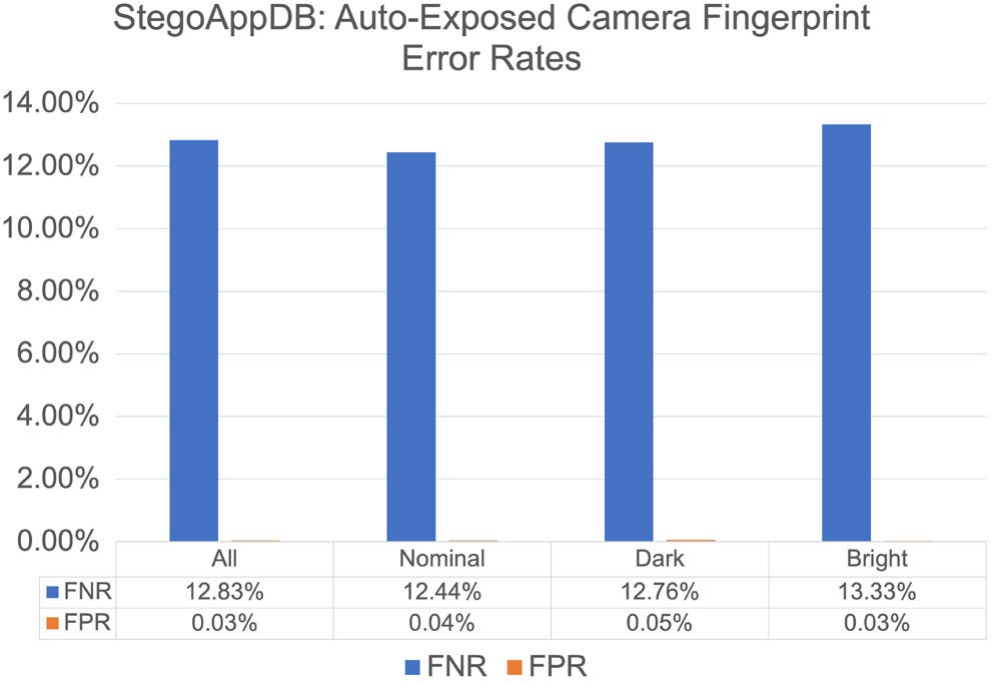
Auto‐Exposed StegoAppDB Camera Fingerprint: False‐negative rates (FNRs) and false‐positive rates (FPRs) averaged for the twenty‐eight auto‐exposed camera fingerprints StegoAppDB cameras with the StegoAppDB questioned images. The results are listed by CCMF label. Note that the *y*‐axis range differs from Figure 6 and Figure 7.

This experiment assumes the exposure settings of the 30 images used to estimate the camera fingerprint are known and were captured with the camera's auto‐exposure settings. The camera fingerprint is estimated for each of the twenty‐eight cameras in StegoAppDB. The questioned images consist of nominal, dark, and bright images where nominal, dark, and bright are the class labels from the CCMF method. Figure 5 presents the FNRs and FPRs separated by the questioned image label of nominal, dark, or bright (left‐hand column).

We evaluate the strength of the evidence that the bright and dark FNR and FPR values are truly different from the nominal questioned images using the hypothesis test outlined in Section IX—Hypothesis Tests. The results of each hypothesis test are listed in Table 3.

**TABLE 3:** StegoAppDB auto‐exposed camera fingerprint: Hypothesis test of difference of proportions of true‐positive rates and true‐negative rates of nominal questioned images compared with dark or bright questioned images. See the text for an interpretation of the strength of the p‐value.

Test #	Alternate hypothesis $\theta_{N}-\theta_{Q}\neq 0$	${\hat{\theta }}_{N}\;=\left(X_{N}/n_{N}\right)$	${\hat{\theta }}_{Q}=\;\left(X_{Q}/n_{Q}\right)$	z‐statistic	p‐value
Difference of Proportions: True‐positive rate (1—FNR)					
1	$\theta_{N}-\theta_{D}\neq 0$	0.8756	0.8724	0.4161	0.678
	(dark questioned images)	(8624/9849)	(1990/2281)		
2	$\theta_{N}-\theta_{B}\neq 0$	0.8756	0.86667	1.76833	0.078
	(bright questioned images)	(8624/9849)	(6786/7839)		

Difference of Proportions: True‐negative rate (1—FPR)					
3	$\theta_{N}-\theta_{D}\neq 0$	0.99965	0.99946	2.17365	0.03
	(dark questioned images)	(265,831/265,923)	(61,554/61,587)		
4	$\theta_{N}-\theta_{B}\neq 0$	0.99965	0.999797	−2.9095	0.0036
	(bright questioned images)	(265,831/265,923)	(211,357/211,400)		

The first hypothesis test has a p‐value of 0.678, which indicates little to no evidence that the TPR is different for dark images than for nominal images. For the second hypothesis test in Table 3, the p‐value of 0.078 indicates little to no evidence that the TPR is different for bright images than for nominal images. Our third hypothesis test using auto‐exposed camera fingerprints has a p‐value of 0.03 indicating little to no evidence that the TNR is different for dark images than for nominal images. Finally, the p‐value of 0.0036 indicates substantial/strong evidence that the TNR is different for bright images than for nominal images.

We remark that the camera model with the highest false‐negative rate by far is the Pixel 2 cameras, which have 2464 of the 2553 false negatives overall. All false negatives for the nominal questioned images are from the Pixel 2 cameras. While the impact of the Pixel 2 images on error rates is interesting, investigating the root cause of these differences is beyond the scope of this paper.

Finally, we implement a power analysis for the hypothesis tests in Table 3 which indicate little to no evidence of differences between the nominal and off‐nominal identification rates. Using the G*Power software [44, 45], we perform a post hoc power analysis to compute achieved power for the TPR hypothesis test for dark questioned images (Test # 1, Table 3), for bright questioned images (Test # 2, Table 3), and for the TNR hypothesis test for dark questioned images (Test # 3, Table 3). The assumptions and results for each test are given in Table 3. For the TPR hypothesis test for dark questioned images, the power level is 0.8085563, which implies a 19.14% chance of failing to conclude there was an effect when there actually was. This is above the standard 0.8 power required for a satisfactory sample size [46], and we must conclude that our sample size is sufficient to observe any effect of dark questioned images on the TPR of the camera identification algorithm when using the auto‐exposed camera fingerprints. The power level for the bright TPR hypothesis test is 0.5005696, and is 0.4978618 for the dark TNR hypothesis test, suggesting that these two sample sizes may be insufficiently large to observe an effect on the error rate.

### 9.2 StegoAppDB: Nominal camera fingerprint

**FIGURE 6:**
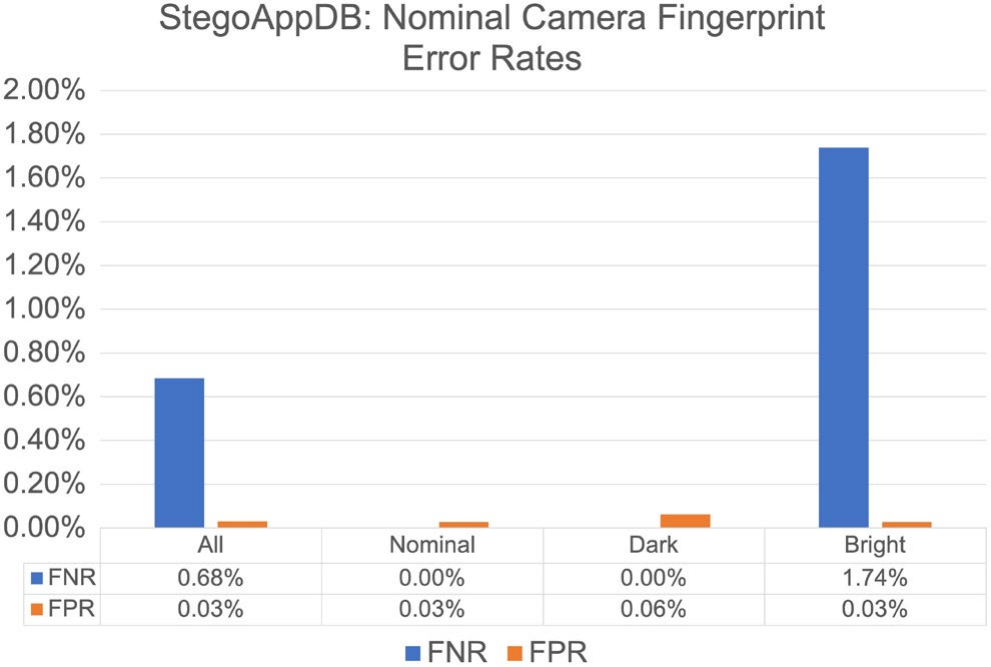
Nominal StegoAppDB Camera Fingerprint: False‐negative rates (FNRs) and false‐positive rates (FPRs) averaged for the twenty‐eight nominal camera fingerprints StegoAppDB cameras with the StegoAppDB questioned images. The results are listed by the CCMF label. Note that the *y*‐axis range differs from Figure 5 and Figure 7.

This experiment assumes the exposure settings of the images used to estimate the camera fingerprint are unknown. We use 30 nominal images for each of the twenty‐eight cameras in StegoAppDB. Figure 6 presents the FNRs and FPRs listed by the questioned image brightness label.

We evaluate the strength of the evidence that the bright and dark FNR and FPR values are truly different from the nominal questioned images using the hypothesis test outlined in Section IX—Hypothesis Tests. The results of each hypothesis test are listed in Table 4.

**TABLE 4:** StegoAppDB nominal camera fingerprint: Hypothesis test of difference of proportions of true‐positive rates and true‐negative rates of nominal questioned images compared with dark or bright questioned images. See the text for an interpretation of the strength of the p‐value.

Test #	Alternate hypothesis $\theta_{N}-\theta_{Q}\neq 0$	${\hat{\theta }}_{N}\;=\left(X_{N}/n_{N}\right)$	${\hat{\theta }}_{Q}\;=\left(X_{Q}/n_{Q}\right)$	z‐statistic	p‐value
Difference of Proportions: True‐positive rate (1—FNR)					
1	$\theta_{N}-\theta_{D}\neq 0$	1	1	N/A	
	(dark questioned images)	(10,246/10,246)	(2418/2418)	(0/0)	
2	$\theta_{N}-\theta_{B}\neq 0$	1	0.9826	13.3980	0.000
	(bright questioned images)	(10,246/10,246)	(8083/8226)		

Difference of Proportions: True‐negative rate (1—FPR)					
3	$\theta_{N}-\theta_{D}\neq 0$	0.99974	0.99937	4.5840	0.0000046
	(dark questioned images)	(276,569/276,642)	(65,245/65,286)		
4	$\theta_{N}-\theta_{B}\neq 0$	0.99974	0.99973	0.03808	0.97
	(bright questioned images)	(276,569/276,642)	(222,043/222,102)		

In particular, note that the p‐value is 0.000 for the difference between the true‐positive rates of the nominal questioned images and the bright questioned images. We can conclude that there is overwhelming evidence that the true‐positive rate of 100% for the nominal questioned images is different from the true‐positive rate of 98.26% for the bright questioned images. Similarly, note that the p‐value is 0.0000046 for the difference between true‐negative rates of the nominal and dark questioned images. Again, we conclude that there is overwhelming evidence that the true‐negative rate for the nominal questioned images is different from the true‐negative rate for the dark questioned images.

Both results support the use of our classification method VII‐B—CCMF Labeling, as statistically significant differences are found between the error rates of nominal question images and both the dark and bright questioned images.

Finally, we implement a power analysis for the hypothesis tests in Table 4 which indicate little to no evidence of differences between nominal and off‐nominal questioned image identification rates. Using the G*Power software [44, 45], we perform a post hoc power analysis to compute the achieved power of the bright questioned image true‐negative rate hypothesis test given an α‐error probability of 0.97. The power level is 0.9817958, which implies a 1.82% chance of failing to conclude there was an effect when there actually was, and is above the 0.8 power typically used to indicate the sample size is large enough to observe an effect [46]. Therefore, we must conclude that our sample size is sufficient to observe any effect of bright questioned images on the TNR. For the hypothesis test of the differences in the true‐positive rates between nominal and dark questioned images, the estimated TPRs are the same, and therefore, we cannot conduct a power analysis. However, we can conclude that there is no evidence that these true‐positive rates differ.

We notice that the false‐negative error rates for StegoAppDB using auto‐exposed camera fingerprints (Figure 5) are much larger than the results for the nominal camera fingerprints (Figure 6). This is due to the Pixel 2 results, which have an FNR of 86.76% for the auto‐exposed camera fingerprint and an FNR of 0.78% for the nominal camera fingerprint. It is beyond the scope of this paper to determine why the Pixel 2 camera model produces auto‐exposed images which match poorly with images of other exposures, but we believe this may be due to proprietary image processing.

### 9.3 Flickr data: Nominal camera fingerprint

**FIGURE 7:**
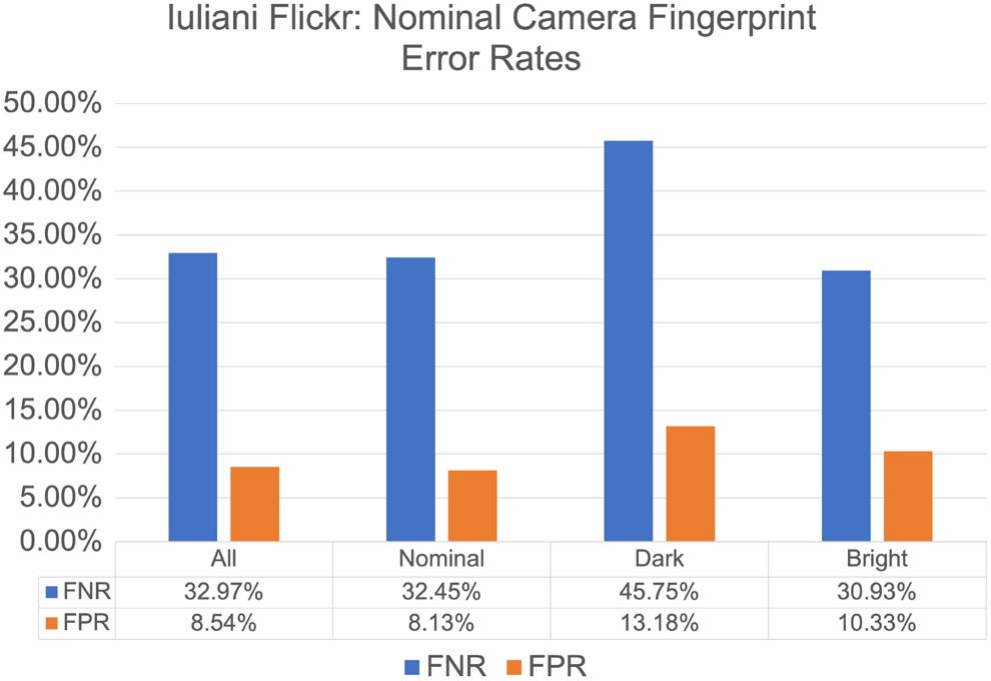
Nominal Iuliani Camera Fingerprint: False‐negative rates (FNRs) and false‐positive rates (FPRs) averaged for the 340 nominal camera fingerprints from Iuliani Flickr cameras with the Iuliani Flickr questioned images. The results are listed by the CCMF label. Note that the *y*‐axis range differs from Figure 5 and Figure 6.

This experiment assumes the exposure settings of the images used to estimate the camera fingerprint are unknown. We use 30 nominal images for each of the 340 of the cameras in the Iuliani Flickr dataset. Figure 7 presents the FNRs and FPRs listed by the questioned image brightness label. We evaluate the strength of the evidence that the bright and dark FNR and FPR values are truly different from the nominal questioned images using the hypothesis test outlined in Section IX—Hypothesis Tests. The results of each hypothesis test are listed in Table 5.

**TABLE 5:** Iuliani Flickr nominal camera fingerprint: Hypothesis test of difference of proportions of true‐positive rates and true‐negative rates of nominal questioned images compared with dark or bright questioned images. See the text for an interpretation of the strength of the p‐value.

Test #	Alternate hypothesis $\theta_{N}-\theta_{Q}\neq 0$	${\hat{\theta }}_{N}=\;\left(X_{N}/n_{N}\right)$	${\hat{\theta }}_{Q}=\;\left(X_{Q}/n_{Q}\right)$	z‐statistic	p‐value
Difference of Proportions: True‐positive rate (1—FNR)					
1	$\theta_{N}-\theta_{D}\neq 0$	0.675499	0.54259	6.4215	0.000
	(dark questioned images)	(7979/11,812)	(293/540)		
2	$\theta_{N}-\theta_{B}\neq 0$	0.675499	0.6907216	−0.6304	0.528
	(bright questioned images)	(7979/11,812)	(268/388)		

Difference of Proportions: True‐negative rate (1—FPR)					
3	$\theta_{N}-\theta_{D}\neq 0$	0.91869	0.86821	118.8684	0.000
	(dark questioned images)	(6,076,944/6,614,788)	(398,630/459,140)		
4	$\theta_{N}-\theta_{B}\neq 0$	0.91869	0.896669	44.478	0.000
	(bright questioned images)	(6,076,944/6,614,788)	(289,886/323,292)		

In particular, note that the p‐value is 0.000 for the difference between the true‐positive rates of the nominal questioned images and the dark questioned images. We can conclude that there is overwhelming evidence that the true‐positive rate for the nominal questioned images is different from the true‐positive rate for the bright questioned images. Similarly, note that the p‐value is 0.000 for the difference between true‐negative rates of the nominal and bright questioned images, and between nominal and dark‐questioned images. Again, we conclude that there is overwhelming evidence that the true‐negative rate for the nominal questioned images is different from the true‐negative rate for the bright or dark questioned images.

These results support the use of our classification method VII‐B—CCMF Labeling, as statistically significant differences are found between the error rates of nominal question images and both the dark and bright questioned images.

We remark that the error rates differ for different cameras, with TPRs ranging from 0% to 100% and TNRs ranging from 6.1% to 99.62% for individual camera fingerprints using all Flickr questioned images. We show select examples of high and low TPR and TNR camera models in Table 6. The initial investigation using these Flickr images also found that error rates differed by camera model, with FPRs calculated for images of the same camera model ranging between 0% and 99.2% [11]. This prior work did not calculate FPRs for images from different camera models or the FNRs for each camera model.

**TABLE 6:** Examples of per model of camera fingerprint results using all Flickr questioned images.

Camera model (# of devices)	TPR (TP/TP + FN)	TNR (TN/TN + FP)
Canon eos 90d	91.25%	45.75%
(2)	(73/80)	(4976/10,877)
Hero8 Black	56.25%	98.83%
(2)	(45/80)	(589/596)
iPhone 11 Pro Max	86.92%	98.92%
(4)	(93/107)	(28,709/29,023)
808 Pureview	100%	23.33%
(5)	(200/200)	(25,379/108,770)
OnePlus a6003	33.63%	94.91%
(8)	(114/339)	(165,191/174,049)

Finally, we implement a power analysis for the hypothesis test in Table 5 which indicates little to no evidence of differences between the nominal and off‐nominal questioned image identification rates. Using the G*Power software [44, 45], we perform a post hoc power analysis and compute the achieved power of the bright questioned image true‐negative rate hypothesis test given an α‐error probability of 0.528. The power level is 0.7609, which implies a 23.91% chance of failing to conclude there was an effect when there actually was. This is slightly below the standard 0.8 power required for a satisfactory sample size [46], so it is possible that our sample size of 388 bright‐questioned images is insufficient to observe any effect of bright questioned images on the TPR of the camera identification algorithm for the Flickr dataset.

Using the hypothesis tests, we are able to confirm that some error rates for dark and bright questioned images are significantly different from the nominal questioned image error rates. The true‐positive rate differs for dark questioned images (Flickr nominal camera fingerprints) and for bright images (StegoAppDB nominal camera fingerprints), and the true‐negative rate differs for dark‐questioned images (both datasets) and for bright‐questioned images (Flickr nominal camera fingerprints). These differences indicate that error rates estimated using nominal questioned images do not accurately represent the error rates for off‐nominal images in the specific cases present here. This is especially problematic for the false‐positive rate (1—TNR), which often provides support in favor of a guilty verdict and can result in wrongful convictions.

## 10 LIMITATIONS

A limitation inherent to the use of images downloaded from Flickr is that the metadata is missing or unreliable, the post‐processing is unknown, the ground‐truth origin of the image is unknown, and the orientation of the camera when the image was collected is unknown. In fact, a recent investigation of the work by Iuliani et al. argues that watermarks, mislabeled devices, and post‐processing by third‐party software likely inflated the original error rate estimates [47]. The images with a different origin or orientation than the other images in the camera fingerprint set could explain the high FNRs for the Flickr data in Figure 7. While we rotate the questioned images and use the maximum PCE score, we cannot feasibly generate camera fingerprints using all possible combinations of rotations of the images. Using 30 camera fingerprint images, the number of fingerprints generated for a single camera using only two of the possible rotations is $2^{30}$, or 1,073,741,824 fingerprints for a single camera.

Another limitation of our work is that we do not produce “within model” false‐positive rate estimates. We assume that the metadata is unreliable and the specific camera model is unknown. Therefore, our FPR estimates are calculated using all cameras in either StegoAppDB or the Flickr dataset, not only the cameras of a specific model. Implementing camera‐model identification could narrow down the cameras to only those with the same camera model as the specific camera, which would change the FPR estimates, as was assumed in [11].

While our post hoc power analyses suggest that an effect may be observed with a larger sample size, we recognize the dependence of these conclusions on the p‐value of the hypothesis test [42, 43]. However, a sample size is a limitation broadly for digital image forensics. The sheer number of images and cameras makes attaining a representative sample a challenge. With a total of 60,354 images across two datasets, our sample size is one of the largest in the field of PRNU source‐camera identification. On the other hand, the camera models represented in our datasets are not the most recent camera models and, therefore, do not implement the technological advancement in the pipeline processing. It is infeasible to maintain data collection at the speed of camera development, but we acknowledge our limitations on more modern cameras.

## 11 DISCUSSION

Recall that our goal for this work is to investigate the effect of the brightness of an image on the error rate for the PRNU source‐camera identification algorithm, not the effect of the source camera on the error rates. Several of our experiments show that error rates for the PRNU source‐camera identification algorithm using bright or dark images can be significantly different than for nominal questioned images. Both the FNR and FPR are significantly different for the dark and bright questioned images than for the nominal questioned images. For the StegoAppDB camera fingerprints from nominal images, the bright questioned image FNR is significantly different from the nominal questioned image FNR (p‐value of 0.000), and the dark questioned image FPR is significantly different from the nominal questioned image FPR (p‐value of 0.0000046). For the Flickr camera fingerprints from nominal images, the dark questioned image FNR is significantly different from the nominal questioned image FNR, and both the dark and bright questioned image FPRs are significantly different from the nominal questioned image FPR (all p‐values of 0.000). Such differences are important when providing error estimates for evidence images in a court of law, and the consistency of the significance of these differences for two datasets provides additional evidence that image brightness impacts error rate estimates. Our CCMF classification method allows a forensic investigator to determine whether a questioned image is bright or dark, regardless of whether the metadata of the image is reliable or not. The significant differences in performance for different types of questioned images further support the use of our classification method to identify potentially problematic images. In fact, our dark and bright ground‐truth images from StegoAppDB were purposefully collected at the extreme ends of their off‐nominal brightness level types, which results in a wide range of brightness levels included in our nominal image set using our CCMF classification method.

In addition to the hypothesis tests between nominal and off‐nominal questioned images, we can determine whether any of our error rates are significantly higher than the large‐scale study [4]. We use the same hypothesis test outlined in Section IX—Hypothesis Tests, but replace the nominal‐questioned image null hypothesis with the results in [4] and use a one‐sided hypothesis test to compute the p‐values given below. [4] uses 2,048,100 PCE scores to estimate an FPR of 0.0024%, indicating approximately 49 images are falsely identified as a match. We note that the false‐positive rates for both bright and dark images from StegoAppDB are significantly higher than the 0.0024% from [4], with z‐scores of 54.12 for the bright images with the nominal camera fingerprints (12.297 with the auto‐exposed camera fingerprints) and 23.26 for the dark images with the nominal camera fingerprints (20.06 with the auto‐exposed camera fingerprints). Z‐scores indicate the number of standard deviations from the population mean. The false‐positive rates are also significantly higher for the images from Flickr, with z‐scores of 462.87 for the bright images and 525.62 for the dark images with the nominal camera fingerprints. Each of the above hypothesis tests with the [4] error rates have a p‐value of 0, which suggests there is overwhelming evidence that the false‐positive rates for bright and dark images are higher than 0.0024%. We chose to focus on false‐positive rates as these are associated with wrongful convictions and are, therefore, the most important error rates to minimize and estimate accurately. Additionally, in our first paper [2] we found that over‐ and under‐exposed questioned images have higher FNRs than auto‐exposed images, which means more images from the suspect camera are missed by the matching algorithm. For each of the three camera fingerprints calculated in this paper, we often find an increased FNR for off‐nominally exposed images, which is in line with our previous results.

Finally, the results using the StegoAppDB camera fingerprints suggest an overarching problem with PRNU source‐camera identification as used in court. Nearly all false negatives in this experiment come from the camera fingerprint of one model: the Pixel 2 with 2464 of the 2560 false negatives. We have four different Pixel 2 cameras and all four have abnormally low true‐positive rates, regardless of the questioned image brightness label. This suggests that one universal error rate estimate is impractical, if not impossible. The FNR using only the four Pixel 2 camera fingerprints with all questioned images is 86.75%, and the FNR using the other 24 camera fingerprints with all questioned images is 0.56%. Similarly, the error rates for the Flickr cameras range from 0% to 100% for TPRs and from 6.1% to 99.62% for TNRs for individual camera fingerprints using all Flickr questioned images. Therefore, it is likely necessary to estimate error rates for each camera model. We leave proposing an alternate standard operating procedure for estimating model‐specific error rates as future work. Additionally, while we would like to investigate the cause of the poor performance of the Pixel 2 camera model, this is outside the scope of this paper. Note that while the Pixel 2 camera model has a poor FNR, the FPR is comparable to the other 9 camera models, with FPRs of 0.019% and 0.035%, respectively.

While our CCMF classification method has a lower accuracy than the machine learning models, our method does not rely on any black box computations and, therefore, is explainable to a jury. Additionally, our classification method is adaptable to new datasets or brightness level definitions, which enables an initial exploration of other methods and definitions for future work. The framework used in this paper for identifying image brightness and determining the impact on camera identification error rates could be adapted to other image features, such as out of focus/blurry images or digital zoom. The primary challenge to replicating this methodology is collecting a varied dataset of controlled images.

## 12 CONCLUSION

Our work clearly demonstrates that the brightness of the questioned image does impact the error rate estimate as given by the PRNU source‐camera‐identification algorithm. In particular, we found that the bright images are more likely to miss being matched correctly with its specific camera, and dark images are more likely to incorrectly match with cameras that did not capture them. Due to error estimates that are clearly impacted by the camera model, we suggest that universal error rates may be infeasible. However, the hypothesis tests show that the differences we observed in errors based on the brightness of the image—nominal, dark, or bright—are significant, suggesting that our classification method is a worthwhile first step toward improving error rate estimates and identifying potentially problematic images when used in the PRNU source‐camera‐identification algorithm. We do not intend the error rates estimated in this work as a replacement to error rates currently used and, instead, aim to show the error rates are different for dark and bright images than for nominal images. Our conclusions are supported by the consistency of several justifications for our labeling of overall image brightness and are also supported by the statistically significant results from our hypothesis tests.

## FUNDING INFORMATION

This work was funded (or partially funded) by the Center for Statistics and Applications in Forensic Evidence (CSAFE) through Cooperative Agreements 70NANB15H176 and 70NANB20H019 between NIST and Iowa State University, which includes activities carried out at Carnegie Mellon University, Duke University, University of California Irvine, University of Virginia, West Virginia University, University of Pennsylvania, Swarthmore College and University of Nebraska, Lincoln.

## CONFLICT OF INTEREST STATEMENT

There are no disclaimers or conflicts of interest.

## HUMAN‐SUBJECTS ASPECT STATEMENT

The Iowa State University Institutional Review Board confirmed that the human‐subjects aspect of this study is exempt; the information collected contains no personally identifiable information and is not intended to contribute to generalizable knowledge.

## Supporting information


Data S1.
