# Supplementary material for: Significance of image brightness levels for PRNU camera identification
Source: J Forensic Sci. 2024 Nov 19;70(1):132–49. doi: 10.1111/1556-4029.15673 (PMC11693528; doi:10.1111/1556-4029.15673)
Supplement: Supplementary file 1 — Data S1. [file JFO-70-132-s001.docx]

# MATHEMATICAL NOTATION

We take our notation from the work presented in (1) which is a heterogeneous image algebra used to represent image processing operations.

Let $X$ be an array of points, often a *rectangular array*, representing an $N\times M$ sensor array:

$$\begin{aligned} X=\left\{ \left( i,j \right):1\leq i\leq N, 1 \leq j\leq M \right\}. \#\left( 1 \right) \end{aligned}$$

Let $\mathbb{V}$ be a set of values for an image, which we refer to as *pixel intensities*. We view a $\mathbb{V}$-valued image with domain $X$ as a function $I\in\mathbb{V}^{X},I:X\to\mathbb{V}$, where

$$\begin{aligned} I=\left\{ \left( \left( i,j \right),I_{ij} \right):\left( i,j \right)\mathbb{\in V} \right\} \#\left( 2 \right) \end{aligned}$$

The domain point $\left( i,j \right)$ is called the *pixel location*. The intensity value of the image $I$ at location $\left( i,j \right)$, denoted $I_{ij}$, is called the *pixel intensity*. Pixel intensities are often 8-bit numbers or 24-bit numbers for color images, but the definitions can be extended to include other value sets, such as $\mathbb{R}$ for the set of real numbers; $\mathbb{Z}_{256}$ for the set of 8-bit integers 0,1,..., 255; or [0,1], the set of real numbers in between 0 and 1, inclusive. The coordinate $\left( \left( i,j \right),I_{ij} \right)$ is called a *pixel* for the image $I$. If $\mathbb{V}$ is a set, we denote the cardinality of $\mathbb{V}$ by $\left| \mathbb{V} \right|$

Now let $I$ be an 8-bit image with intensities between 0 and 255. For $I$, we define the normalized histogram or *probability mass function* (PMF) $h^{I}$ representing the normalized frequency of pixel intensities in the image by

$$\begin{aligned} h^{I}=\left\{ \left( k,h^{I}\left( k \right) \right):0\leq k\leq255, 0\leq h^{I}\left( k \right)=\frac{\left| \left\{ \left( i,j \right):I_{ij}=k \right\} \right|}{NM}\leq1 \right\}. \#\left( 3 \right) \end{aligned}$$

Define the *cumulative mass function* (CMF) for $I$, denoted $H^{I}$, as:

$H^{I}=\left\{ \left( k,H^{I}\left( k \right) \right):H^{I}\left( k \right)=\sum_{I=0}^{k} h^{I}\left( I \right),0\leq k\leq255 \right\}$.

Given an image, $I$, with cumulative histogram $H^{I}$, we are interested in the descriptive statistic ${1-H}^{I}$ called the *complementary cumulative mass function* (CCMF) or *survivor function*, that we denote by $S^{I}$:

$$\begin{aligned} S^{I}=1-H^{I}=\left\{ \left( k,S^{I}\left( k \right) \right):S^{I}\left( k \right)=1-H^{I}\left( k \right), 0\leq k\leq255 \right\}. \#\left( 4 \right) \end{aligned}$$

We note that $S^{I}$ is a decreasing function in $k$ and that for pixel intensity $k$, the value of $S^{I}\left( k \right)$ is the fraction of pixel locations in the image $I$ whose intensities are greater than or equal to $k+1$.

# EXPOSURE VALUE

The $EV$ is expressed by the equation (2):

$$\begin{aligned} EV=\log_{2} \left( \frac{N^{2}}{t} \right), \#\left( 5 \right) \end{aligned}$$

where $N$ is from the $f$-number: $\frac{f}{N}$ and $t$ is the exposure time in seconds.

The exposure value $EV$ can also be expressed as

$$\begin{aligned} EV=\log_{2} \left( \frac{LS}{K} \right), \#\left( 6 \right) \end{aligned}$$

where $L$ is the average scene luminance, $S$ is the ISO value, and $K$ is a calibration constant for the reflected-light meter of the camera. Most tables for $EV$ assume an ISO value of 100. $EV$ for other ISO values are derived from (5) and (6). We subscript $EV$ with its ISO value; thus ${EV}_{400}$ means the ISO is 400.

To derive the quantitative relationship between image exposure types for our data, let $I_{A}$ (auto-exposed), $I_{U}$ (under-exposed), and $I_{O}$ (over-exposed) denote the three photos taken of one scene and within several seconds of each other in StegoAppDB. Let $I_{A}$ have ISO value $S_{A}$ and exposure time $t_{A}$. We assume that ${EV}_{S_{A}}$ is the baseline exposure value for this group of three pictures and that luminance $L$ is approximately constant over the time span of acquisition for all three images. The calculated exposure settings for images $I_{U}$ and $I_{O}$ are given in Section VI-A - StegoAppDB Details.

Substituting the ISO ($S_{A}$) and exposure time ($t_{A}$) of $I_{A}$ into (5) and (6), we have

$$\begin{aligned} \frac{N^{2}}{t_{A}}=\frac{L*S_{A}}{K}. \#\left( 7 \right) \end{aligned}$$

Using ${EV}_{100}$ as the exposure value for the auto-exposed scene acquired at ISO = $100$:

$$\frac{N^{2}}{t_{A}}=\frac{L*S_{A}}{k}=\frac{L*100}{k}*\frac{S_{A}}{100}$$

$$\begin{aligned} {EV}_{S_{A}}={EV}_{100}+\log_{2} \left( \frac{S_{A}}{100} \right). \#\left( 8 \right) \end{aligned}$$

Similarly, for $I_{U}$ we have

$$\begin{aligned} {EV}_{S_{U}}={EV}_{100}+\log_{2} \left( \frac{S_{U}}{100} \right). \#\left( 9 \right) \end{aligned}$$

Since we know that $S_{U}=0.5*S_{A}$, we substitute into (9) to calculate ${EV}_{S_{U}}$:

$$\begin{aligned} {EV}_{S_{U}}={EV}_{100}+\log_{2} \left( \frac{0.5*S_{A}}{100} \right) \#\left( 10 \right) \end{aligned}$$

$$\begin{aligned} ={EV}_{100}+\log_{2} \left( \frac{S_{A}}{100} \right)-1 \#\left( 11 \right) \end{aligned}$$

$$\begin{aligned} {EV}_{S_{U}}<{EV}_{S_{A}}. \#\left( 12 \right) \end{aligned}$$

Equation (12) states that the exposure value for image $I_{U}$ is less than the exposure value for auto-exposed image $I_{A}$. Since $I_{U}$ is not auto-exposed, it appears visually darker or “under-exposed” relative to the auto-exposed image $I_{A}$.

By a similar process:

$$\begin{aligned} {EV}_{S_{O}}={EV}_{100}+\log_{2} \left( \frac{S_{A}}{100} \right)+\log_{2} \left( 3 \right) \#\left( 13 \right) \end{aligned}$$

$$\begin{aligned} {EV}_{S_{O}}>{EV}_{S_{A}}. \#\left( 14 \right) \end{aligned}$$

Equation (14) states that the exposure value for image $I_{O}$ is greater than the exposure value for image $I_{A}$. Since $I_{O}$ is not auto-exposed, it appears lighter or “over-exposed” relative to the auto-exposed image $I_{A}$. These relations hold for all triples of auto-, under-, and over-exposed images from StegoAppDB.

# MACHINE LEARNING MODELS

# *Decision Trees*

The CCMFs for all auto-, under-, and over-exposed images were labeled with a one-hot vector. Then, we split the data into two equal-sized and randomly drawn sets, a training and a test set (i.e., 50% of the data is used for training and 50% of the data is used for testing). The process of partitioning the data is repeated for each decision tree generated. A total of three decision trees were used: one using auto- and under-exposed images, one using auto- and over-exposed images, and one using auto-, under-, and over-exposed images. Each tree was generated using sci-kit learn Decision Tree Classifier trained with the default parameters (3) (4). While the structure is different for the three trees (varying between three and twenty-six leaf notes), each has an accuracy of 100%. The primary concern for each of these decision trees is overfitting.

*Random Forest*

Again, each image CCMF is labeled with a one-hot vector. Once the images are all labeled, the data is split randomly into training and test sets, with half of the images used to train the model and half of the images reserved for testing. The process of partitioning the data into training and test sets is repeated for each random forest generated. A total of three random forests were tested: one using auto- and under-exposed images, one using auto- and over-exposed images, and one using auto-, under-, and over-exposed images. Each tree was generated using the sci-kit learn Random Forest Classifier trained with the default parameters (5). Each of the three random forests have an accuracy of 100%, but our primary concern is still overfitting the training data.

*Support Vector Machines (SVMs)*

The CCMFs for all auto-, under-, and over-exposed images were labeled nominal, dark, and bright, respectively. Again, the images are split into training and test sets, with half of the images used to train the model and half of the images reserved for testing. The process of partitioning the data into training and test sets is repeated for each SVM generated. A total of three SVMs were tested: one using auto- and under-exposed images, one using auto- and over-exposed images, and one using auto-, under-, and over-exposed images. Each SVM was generated using sci-kit learn SVC trained with the default parameters and probability set to `True' (6). Each of the three support vector machines has an accuracy of 100%. Again, over-fitting is a concern.

*Convolutional Neural Networks (CNNs)*

Our final machine learning classification method uses convolutional neural networks (CNNs). For the first CNN, all auto-, under-, and over-exposed images were read into Python with the validation split set to 50%, image size of $32 \times32$, and the images cropped to the aspect ratio set to True (7). Validation split indicates the fraction of data reserved for validation, image size is the size that an image is resized to, specified as height X width. The final setting indicates that the images will be resized to the aspect ratio without distortion. We use a sequential model (8) with eight layers (three convolutional layers (9), two max pooling layers (10), a flattening layer (11), and two densely-connected layers (12)). The eight layers of each of our CNNs are as follows: 1) a 2D convolutional layer, 2) a max pooling 2D layer, 3) another 2D convolutional layer, 4) another max pooling 2D layer, 5) the final 2D convolutional layer, 6) a flattening layer, 7) a densely connected CNN layer, and 8) a final densely connected CNN layer. We use a stochastic-gradient-descent optimizer and train the model for ten epochs. The tenth epoch had the highest accuracy value of 96.50%.

The second CNN used followed the same structure and settings as the first, except the images were resized to $128 \times128$. The ninth epoch had the highest accuracy value (96.36%), while the tenth epoch had an accuracy of 61.71%. Our concern with the CNNs is not only accuracy, but variability in results with different arbitrary training parameters.

# DETERMINING CCMF-CLASSIFICATION PARAMETERS

First, we analyze the differences between the CCMFs of the auto- and under-exposed images in StegoAppDB. Recall $\mathcal{A}$ is the set of 2,836 auto-exposed images and $\mathcal{U}$ is the set of 2,836 under-exposed images.

Let $\mathcal{C}^{\mathcal{A}}$be the set of CCMFs from all images in $\mathcal{A}$, and let $\mathcal{C}^{\mathcal{U}}$ be the set of CCMFs from all images in $\mathcal{U}$:

$$\begin{aligned} \mathcal{C}^{\mathcal{A}}=\left\{ S^{I}:I\in\mathcal{A} \right\} \#\left( 15 \right) \end{aligned}$$

$$\begin{aligned} \mathcal{C}^{\mathcal{U}}=\left\{ S^{I}:I\in\mathcal{U} \right\} \#\left( 16 \right) \end{aligned}$$

Note that for any image $I\in\mathcal{A\cup U}$ the range of the function $S^{I}$ is the unit interval [0, 1] and that for a fixed gray value $k$, $S^{I}\left( k \right)=r, 0\leq r\leq1$. In particular, due to the definition of $S^{I}$, $S^{I}\left( k \right)=r$ means that for the image $I$, the fraction of pixels whose intensities are greater than or equal to $k$ is $\left( 100*r \right)\%$. Also note that $\left( k,S^{I}\left( k \right) \right)$ is a point in $\mathbb{R}^{2}$ along the line $x=k$, and that for a fixed $k$ each image $I\in\mathcal{A\cup U}$ is represented by its value $S^{I}\left( k \right)$ along the line $x=k$ by the point $\left( k,S^{I}\left( k \right) \right)$.

We now fix $k$ and construct the set of CCMF values at $k$ for all images in $\mathcal{A}$ and in $\mathcal{U}$:

$$\begin{aligned} \mathcal{C}^{\mathcal{A}}\left( k \right)=\left\{ \left( k,S^{I}\left( k \right) \right):I\in\mathcal{A} \right\} \#\left( 17 \right) \end{aligned}$$

$$\begin{aligned} \mathcal{C}^{\mathcal{U}}\left( k \right)=\left\{ \left( k,S^{I}\left( k \right) \right):I\in\mathcal{U} \right\} \#\left( 18 \right) \end{aligned}$$

and choose a value $f$, $0\leq f\leq1$. The value $f$ partitions the set of all points $\mathcal{C}^{\mathcal{A}}\left( k \right)\cup\mathcal{C}^{\mathcal{U}}\left( k \right)$ into two new sets - one set whose CCMF values $S^{I}\left( k \right)$ are less than $f$, and the other set whose CCMF values $S^{I}\left( k \right)$ are greater than or equal to $f$:

$$\begin{aligned} L_{<f}\left( k \right)=\left\{ \left( k,S^{I}\left( k \right) \right):S^{I}\left( k \right)<f, I\in\mathcal{A\cup U} \right\} \#\left( 19 \right) \end{aligned}$$

$$\begin{aligned} L_{\geq f}\left( k \right)=\left\{ \left( k,S^{I}\left( k \right) \right):S^{I}\left( k \right)\geq f, I\in\mathcal{A\cup U} \right\}. \#\left( 20 \right) \end{aligned}$$

Note that it is likely that $L_{<f}\left( k \right)$ contains a mix of points from both image sets $\mathcal{A}$ and $\mathcal{U}$, and that the same holds true for $L_{\geq f}\left( k \right)$. For this $k$, we compute the accuracy for this fraction threshold $f$ to separate the auto-exposed images from $\mathcal{A}$ into $L_{\geq f}\left( k \right)$ and the under-exposed images from $\mathcal{U}$ into $L_{<f}\left( k \right)$. We do this by simply counting the number of points $\left( k,S^{I}\left( k \right) \right)$ in $L_{\geq f}\left( k \right)$ where $I\in\mathcal{A}$, and counting the number of points $\left( k,S^{I}\left( k \right) \right)$ in $L_{<f}\left( k \right)$ where $I\in\mathcal{U}$.


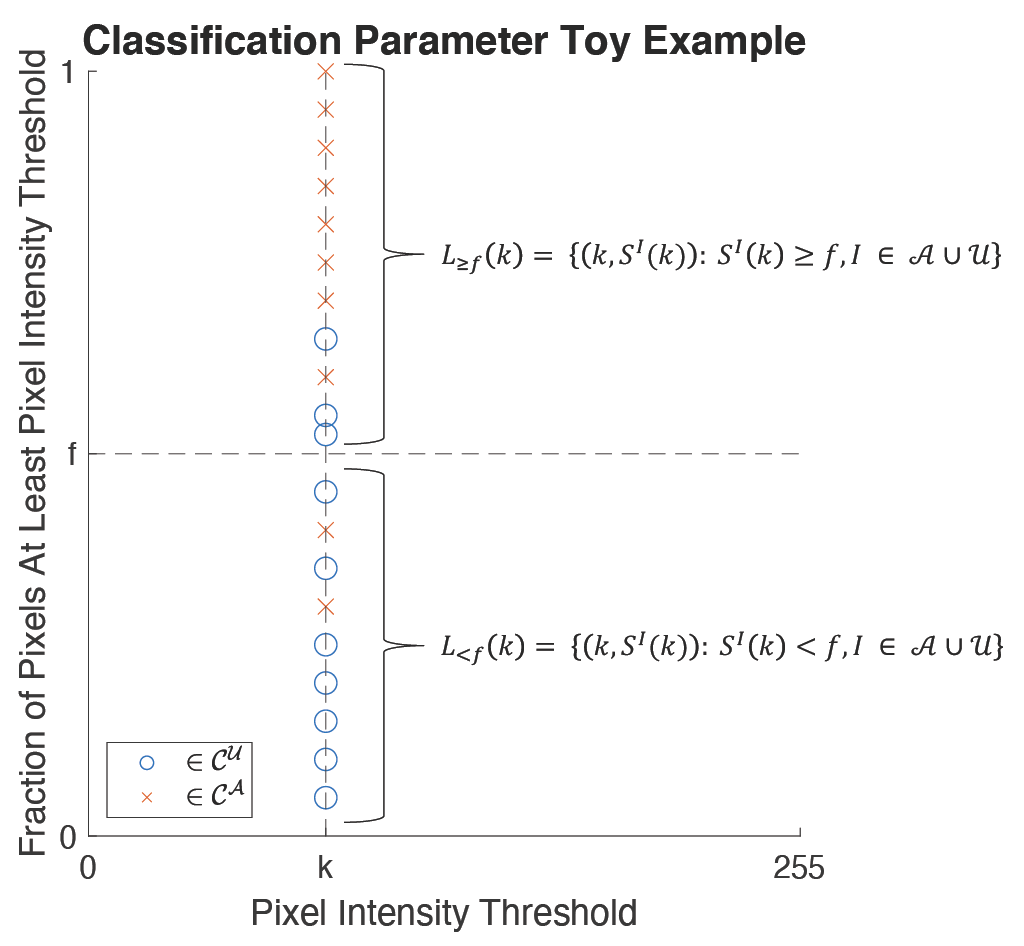


Figure 1. This toy example uses 10 images in $\mathcal{C}^{\mathcal{A}}$ (two below the threshold $f$) and 10 images in $\mathcal{C}^{\mathcal{U}}$ (three above the threshold $f$). For parameters $f$ and $k$, this example has an accuracy calculated: ${Acc}_{D}=\frac{8+7}{20}=75\%$.

The labels for images in the set $L_{\geq f}\left( k \right)$ is “nominal,” and the label for images in the set $L_{<f}\left( k \right)$ is “dark,” and any images in the wrong set are errors, since auto-exposed images are assumed to be nominal and under-exposed images are assumed to be dark.

$$\begin{aligned} L_{<f}\left( k \right)=\left\{ \left( k,S^{I}\left( k \right) \right):S^{I}\left( k \right)<f,I\in\mathcal{A} \right\}\cup\left\{ \left( k,S^{I}\left( k \right) \right):S^{I}\left( k \right)<f, I\in\mathcal{U} \right\}=L_{<f}^{\mathcal{A}}\left( k \right)\cup L_{<f}^{\mathcal{U}}\left( k \right) \#\left( 21 \right) \end{aligned}$$

$$\begin{aligned} L_{\geq f}\left( k \right)=\left\{ \left( k,S^{I}\left( k \right) \right):S^{I}\left( k \right)\geq f,I\in\mathcal{A} \right\}\cup\left\{ \left( k,S^{I}\left( k \right) \right):S^{I}\left( k \right)\geq f, I\in\mathcal{U} \right\}=L_{\geq f}^{\mathcal{A}}\left( k \right)\cup L_{\geq f}^{\mathcal{U}}\left( k \right). \#\left( 22 \right) \end{aligned}$$

Then the dark accuracy for labeling the set $\mathcal{A\cup U}$ using $k$ and $f$ is:

$$\begin{aligned} {Acc}_{D}\left( k,f \right)=\frac{\left| L_{\geq f}^{\mathcal{A}}\left( k \right) \right|+\left| L_{<f}^{\mathcal{U}}\left( k \right) \right|}{\left| \mathcal{A} \right|+\left| \mathcal{U} \right|}. \#\left( 23 \right) \end{aligned}$$

See Fig. 1 for a toy example of this calculation.

For the over-exposed images, we follow a similar process:

$$\begin{aligned} \mathcal{C}^{\mathcal{A}}=\left\{ S^{I}:I\in\mathcal{A} \right\}, \#\left( 24 \right) \end{aligned}$$

$$\begin{aligned} \mathcal{C}^{\mathcal{O}}=\left\{ S^{I}:I\in\mathcal{O} \right\}. \#\left( 25 \right) \end{aligned}$$

The interpretation of the two parameters $k$ and $f$ remain the same: Fix an intensity value $k$, $k\in\left\{ 0, 1, \ldots, 255 \right\}$, and construct the four sets $L_{<f}^{\mathcal{O}}\left( k \right)$, $L_{\geq f}^{\mathcal{O}}\left( k \right)$, $L_{<f}^{\mathcal{A}}\left( k \right)$, and $L_{\geq f}^{\mathcal{A}}\left( k \right)$. Calculate the bright accuracy for labeling the set $\mathcal{A\cup O}$ using $k$ and $f$ as:

$$\begin{aligned} {Acc}_{B}\left( k,f \right)=\frac{\left| L_{<f}^{\mathcal{A}}\left( k \right) \right|+\left| L_{\geq f}^{\mathcal{O}}\left( k \right) \right|}{\left| \mathcal{A} \right|+\left| \mathcal{O} \right|}. \#\left( 26 \right) \end{aligned}$$

# RESULTS TABLES

The following tables correspond to Fig. 6, Fig. 7, and Fig. 8:

| **StegoAppDB: Auto-Exposed Camera Fingerprint** | | |
| --- | --- | --- |
| **Questioned Image**  **Brightness Label** | **FNR**  **(FN/FN+TP)** | **FPR**  **(FP/FP+TN)** |
| **All** | 12.83%  (2,560/19,960) | 0.033%  (178/538,920) |
| **Nominal** | 12.44%  (1,225/9,849) | 0.035%  (92/265,923) |
| **Dark** | 12.76%  (291/2,281) | 0.054%  (33/61,587) |
| **Bright** | 13.33%  (1,044/7,830) | 0.025%  (53/211,410) |

***Table 1:*** *False Negative Rates (FNRs) and False Positive Rates (FPRs) averaged for the twenty-eight auto-exposed camera fingerprints StegoAppDB cameras with the StegoAppDB questioned images. The results are listed by CCMF label.*

| **StegoAppDB: Nominal Camera Fingerprint** | | |
| --- | --- | --- |
| **Questioned Image**  **Brightness Label** | **FNR**  **(FN/FN+TP)** | **FPR**  **(FP/FP+TN)** |
| **All** | 0.6845%  (143/20,890) | 0.0307%  (173/564,030) |
| **Nominal** | 0%  (0/10,246) | 0.0264%  (73/276,642) |
| **Dark** | 0%  (0/2,418) | 0.0628%  (41/65,286) |
| **Bright** | 1.7384%  (143/8,226) | 0.0266%  (59/222,102) |

***Table 2:*** *False Negative Rates (FNRs) and False Positive Rates (FPRs) averaged for the twenty-eight nominal camera fingerprints StegoAppDB cameras with the StegoAppDB questioned images. The results are listed by the CCMF label.*

| **Iuliani Flickr: Nominal Camera Fingerprint** | | |
| --- | --- | --- |
| **Questioned Image**  **Brightness Label** | **FNR**  **(FN/FN+TP)** | **FPR**  **(FP/FP+TN)** |
| **All** | 32.97%  (4,200/12,740) | 8.54%  (631,760/7,397,220) |
| **Nominal** | 32.45%  (3,833/11,812) | 8.13%  (537,844/6,614,788) |
| **Dark** | 45.75%  (247/540) | 13.18%  (60,510/459,40) |
| **Bright** | 30.93%  (120/388) | 10.33%  (33,406/323,292) |

***Table 3:*** *False Negative Rates (FNRs) and False Positive Rates (FPRs) averaged for the 340 nominal camera fingerprints from Iuliani Flickr cameras with the Iuliani Flickr questioned images. The results are listed by the CCMF label.*

# Bibliography

| 1. | Ritter GX, Wilson JN, Davidson JL. Image Algebra: An Overview. Comput Vis Graph Image Process. 1990; 49: 297-331. doi: 10.1016/0734-189X(90)90106-6. |
| --- | --- |
| 2. | Lee HC. Introduction to Color Imaging Science: Cambridge University Press; 2005. |
| 3. | Pedregosa F, Varoquaux G, Gramfort A, Michel V, Thirion B, Grisel O, et al. Scikit-learn: Machine Learning in Python. Journal of Machine Learning Research. 2011; 12: 2825-2830. |
| 4. | Scikit Learn. Scikit Learn. [Online]. [cited 2024 February. Available from: <https://scikit-learn.org/stable/modules/generated/sklearn.tree.DecisionTreeClassifier.html>. |
| 5. | Scikit Learn. Scikit Learn. [Online]. [cited 2024 February. Available from: <https://scikit-learn.org/stable/modules/generated/sklearn.ensemble.RandomForestClassifier.html>. |
| 6. | Scikit Learn. Scikit Learn. [Online]. [cited 2024 February. Available from: <https://scikit-learn.org/stable/modules/generated/sklearn.svm.SVC.html>. |
| 7. | TensorFlow. TensorFlow. [Online].; 2024 [cited 2024 February. Available from: <https://www.tensorflow.org/api_docs/python/tf/keras/preprocessing/image_dataset_from_directory>. |
| 8. | TensorFlow. TensorFlow. [Online].; 2024 [cited 2024 February. Available from: <https://www.tensorflow.org/api_docs/python/tf/keras/Sequential>. |
| 9. | TensorFlow. TensorFlow. [Online].; 2024 [cited 2024 February. Available from: <https://www.tensorflow.org/api_docs/python/tf/keras/layers/Conv2D>. |
| 10. | TensorFlow. TensorFlow. [Online].; 2024 [cited 2024 February. Available from: <https://www.tensorflow.org/api_docs/python/tf/keras/layers/MaxPool2D>. |
| 11. | TensorFlow. TensorFlow. [Online].; 2024 [cited 2024 February. Available from: <https://www.tensorflow.org/api_docs/python/tf/keras/layers/Flatten>. |
| 12. | TensorFlow. TensorFlow. [Online].; 2024 [cited 2024 February. Available from: <https://www.tensorflow.org/api_docs/python/tf/keras/layers/Dense>. |
